# Epidemiological and Clinical Characteristics of COVID-19 in Children: A Systematic Review and Meta-Analysis

**DOI:** 10.3389/fped.2020.591132

**Published:** 2020-11-02

**Authors:** Bingbing Li, Shan Zhang, Ruili Zhang, Xi Chen, Yong Wang, Changlian Zhu

**Affiliations:** ^1^Henan Key Laboratory of Child Brain Injury, Department of Pediatrics, The Third Affiliated Hospital and Institute of Neuroscience of Zhengzhou University, Zhengzhou, China; ^2^Center for Brain Repair and Rehabilitation, Institute of Neuroscience and Physiology, Sahlgrenska Academy, University of Gothenburg, Gothenburg, Sweden; ^3^Department of Women's and Children's Health, Karolinska Institutet, Stockholm, Sweden

**Keywords:** SARS-CoV-2, epidemiology, clinical characteristics, children, meta-analysis

## Abstract

Given the relatively low rate and limited publicly available data regarding children with SARS-CoV-2 infection, this knowledge gap should be addressed with urgency. This systematic review with meta-analysis aimed to evaluate the epidemiological spectrum and clinical characteristics of children infected with SARS-CoV-2. Relevant international and Chinese public databases were systematically searched to identify all case studies from January 1, 2020 to May 7, 2020. This study consisted of 96 studies involving 7004 cases. The mean age of pediatric cases was 6.48 years (95% CI 52.0–77.5), 90% had household contact, and 66% presented with mild to moderate clinical syndromes. The main symptoms were fever (47%, 95% CI 41–53%) and cough (42%, 95% CI 36–48%). About 23% of children were asymptomatic, 27% had comorbidity, and 29% had a co-infection. The pooled mean incubation period was 9.57 days (95% CI 7.70–11.44). The shedding of SARS-CoV-2 in the upper respiratory tract lasted 11.43 days, and 75% of patients had virus particles in their stool. A total of 34% of the children had neutropenia and 26% had lymphocytosis. Interferon-alpha (81%) was the most commonly used antiviral drug in the children. The discharge and death rates were 79 and 1%. In conclusion, the transmissibility of pediatric COVID-19 should be not ignored because of the relatively long incubation period, shedding duration, and mild clinical syndromes.

## Introduction

A cluster of patients with severe pneumonia of unknown etiology appeared in China in December 2019, leading to the discovery of an emerging infectious virus (1). The causative agent was isolated and named 2019 novel coronavirus (2019-nCoV) and then officially called severe acute respiratory syndrome coronavirus 2 (SARS-CoV-2). Coronavirus disease 2019 (COVID-19) caused by SARS-CoV-2 swept through mainland China and spread around the world, with a 5.9% case fatality rate (2).

All ages are susceptible to SARS-CoV-2. However, the proportion of confirmed cases in children has been relatively small. The largest epidemiological survey in China showed that 2.2% of children were affected (3), and this number was 1.7% in the USA (4). For this reason, most of the guidelines published so far are more appropriate for adults than for children. Several systematic reviews with simple meta-analysis have been reported, but these have mainly focused on the clinical characteristics of pediatric COVID-19 (5–7), and data on the transmissibility, rate of viral coinfections, and treatments of pediatric COVID-19 are still lacking. Asymptomatic transmission is the Achilles' heel of COVID-19 pandemic control, as reported by Gandhi et al. (8), but the number of asymptomatic infections in children is unknown. Furthermore, children are often unable to clearly describe their health status or history of exposure, posing serious challenges to protecting, diagnosing, and treating this population. Thus, we performed a systematic review and meta-analysis of the published literature to summarize the current knowledge of COVID-19 in children with respect to epidemiology, clinical characteristics, rate of viral coinfections, and outcomes.

## Methods

### Protocol and Registration

This systematic review and meta-analysis was conducted according to the PRISMA guidelines (9) and is registered on Prospective Register of Systematic Reviews (Registration No. CRD42020180126).

### Search Strategy

We performed a comprehensive systematic literature search in key electronic databases, including PubMed/Medline, Web of Science, OVID, medRxiv, Wan Fang Data, and China National Knowledge Infrastructure (CNKI), from January 1, 2020 to May 7, 2020, to identify all case studies. The following search terms were used in all possible combinations: “novel coronavirus or COVID-19 or 2019-nCoV or SARS-CoV-2 or novel coronavirus pneumonia” and “pediatrics or pediatrics or neonate or newborn or infant or children or adolescence or teenagers” (see Supplementary Table 1 for the search strategy). In addition, the reference lists of all known primary and review articles were scrutinized to identify cited articles not captured by the electronic searches. Google Scholar was also searched manually for possible missing articles. Moreover, the CDC and WHO portals of the National Public Health Institute were evaluated.

### Inclusion Criteria and Study Selection

Studies were deemed eligible for inclusion if they (1) were case reports, case series (at least three patients), or observational studies; (2) were cases of laboratory-confirmed COVID-19 patients younger than 18 years old; and (3) reported clear, extractable data for epidemiological, clinical, laboratory, and radiological characteristics, treatments, and outcomes. Correspondences or letters fulfilling the aforementioned criteria were also included. The severity of COVID-19 was defined according to the largest cohort reported of >44,000 persons with COVID-19 issued from the Chinese CDC (3), which showed that illness severity can range from mild to critical, including asymptomatic infection.

The exclusion criteria were as follows: (1) repeated calculations or duplicate studies; (2) reporting cases with incomplete information; (3) studies including both adults and children but not presenting sufficient data for children; (4) adult-only or abstract-only studies; (5) review articles, meta-analyses, perspectives, comments, consensus documents, opinion articles, and letters not presenting original data; (6) publications with suspected but not laboratory-confirmed cases; (7) articles written in languages other than English or Chinese.

### Study Selection and Risk of Bias Assessment

The titles and abstracts of each article identified in the search were assessed for eligibility according to the inclusion criteria by two independent reviewers (R.Z. and X.C.). Full-text articles were obtained for evaluation. Any discrepancies during the selection assessment were resolved by discussion and consensus. All articles published in Chinese were assessed by a medical professional fluent in both Chinese and English. If the patients came from the same hospital with overlapping cases, we only selected the publication containing the greatest number of cases. The process of study selection is reported here in full in the final report with a PRISMA flow diagram.

The risk of bias for eligible case–control and cohort studies was assessed according to the Newcastle–Ottawa Scale, and a score >4 was considered high quality. For cross-sectional studies, we used the Appraisal tool for Cross Sectional Studies (10). For observational case series studies, we used the Quality Appraisal of Case Series Studies Checklist of the IHE (11). Both assess bias according to 20 criteria, respectively, and for each “Yes” item the score is 1, and for each “partial or no” item the score is 0. The higher the total score, the lower the risk of bias.

### Data Extraction

Two reviewers (B.L. and S.Z.) extracted the data independently with a standardized data collection form, including (1) basic information, (2) demographic information (age and sex), (3) clinical symptoms, (4) laboratory blood tests, (5) chest CT findings, and (6) therapies and prognoses.

For dichotomous outcomes, we extracted the number of events and total participants per group. For continuous outcomes, we extracted means, SDs, and the total participants in each group. If means and SDs were not reported, we calculated them from the reported indicators (12). If data were missing or reported in an unusable way, we excluded the study from the meta-analysis and report the findings descriptively.

### Data Analysis

Observational studies with at least three patients with available data were included for meta-analysis. Case reports were not included for the meta-analysis, but we present the data with descriptive statistics. For dichotomous data, we performed a single-arm meta-analysis of proportions (and 95% CIs) using STATA 12.0. The meta-analysis was performed using the “metaprop” program in which all original data included in the literature were first transformed by the double arcsine method to make them conform to normal distributions. For continuous data, we performed a meta-analysis of continuous variables, calculating the effect sizes with 95% CIs. Heterogeneity of the studies was assessed using the Cochran *Q*-test and Higgins' *I*^2^ statistic expressed as percentages. For the χ^2^-test, significant heterogeneity among the studies was indicated with a Cochran's Q *p*-value of <0.10. Values of 25, 50, and 75% for *I*^2^ were considered low, medium, and high levels of heterogeneity, respectively. Data were pooled using random-effects models due to clinical and methodological heterogeneity in the study designs and features of the participants.

## Results

### Outcome of the Electronic Search

**Figure 1:**
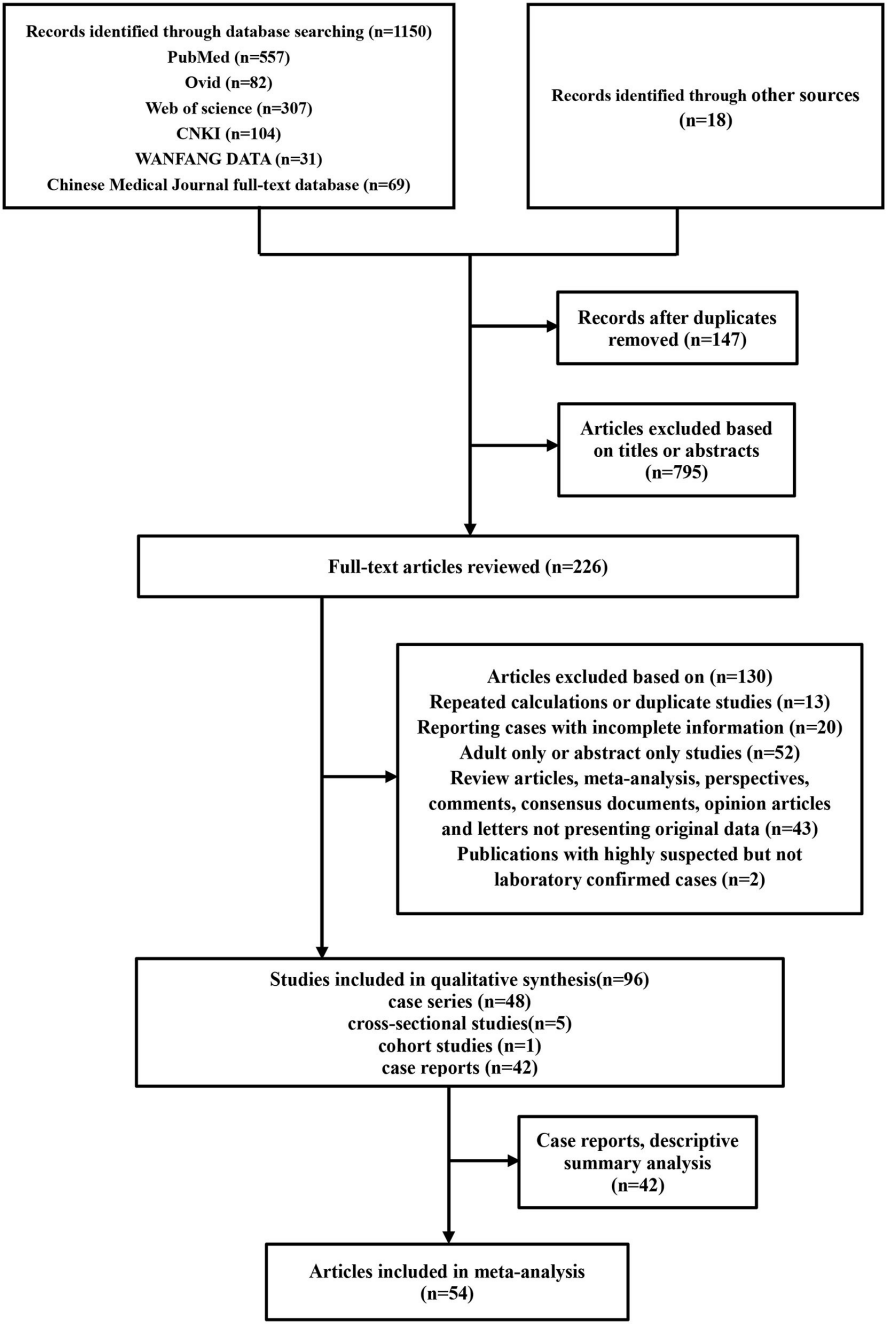
Flowchart depicting the literature search and selection strategy. After applying the inclusion and exclusion criteria, a total of 54 articles were included in the final meta-analysis.

A total of 1,150 articles were retrieved. After removing duplicates and excluding irrelevant articles, 226 full-text articles were assessed. Eighteen records were identified through manual searches of Google Scholar. Ninety-six were ultimately included for qualitative analysis, including 54 for quantitative meta-analysis and 42 case reports for descriptive analysis (Figure 1).

### Characteristics of the Included Studies and Demographic Characteristics

Fifty-four eligible studies with 6,951 COVID-19 pediatric patients were included. The studies were published between January 1, 2020 and May 7, 2020, among which 36 (66.7%) were written in English and 18 in Chinese. Most studies reported COVID-19 cases from China, with the exception of five studies from the USA, Spain, Malaysia, and multiple countries. The total number of pediatric cases from China was 4280 (61.6%). The quality scores of the included studies showed low risk of bias (Supplementary Table 2). Most included studies were retrospective case series, except five cross-sectional and one cohort study (Supplementary Tables 3–7). There were 95 variables included in this analysis. The number of cases enrolled in each study ranged from four to 2,572, and the age was 1 day to 18 years. The mean age of patients across 49 studies was 6.48 years (95% CI 5.20–7.75). In particular, 19% (95% CI 15–22%) of children were <1 year, 34% (95% CI 21–48%) were 1–5 years old, and 57% (95% CI 52–62%) were older than 5 years. The ratio of boys to girls was 1.31 (95% CI 55–59%).

### Epidemiological Characteristics

Family cluster exposure was reported in 39 studies, which involved 90% (95% CI 85–94%) of the children. In 34 studies, 65% (95% CI 49–80%) of cases had traveled to or were residents of Hubei Province. In 24 studies, 82% (95% CI 74–89%) of cases had contact with confirmed or suspected adult COVID-19 patients. Eleven studies reported 23% (95% CI 5–47%) of cases had unclear exposure history. There was significant medium to high heterogeneity (Cochran's *Q*) in the estimates of clinical symptoms among the examined studies (*p* < 0.001) with an *I*^2^ index of 72.23–97.95%.

The mean incubation period was 9.57 days (95% CI 7.70–11.44) based on eight studies. The pooled mean time from onset of symptoms to diagnosis was 3.07 days (95% CI 2.54–3.60) according to 15 studies. The pooled mean time from close contact to diagnosis of COVID-19 was 10.8 days (95% CI 8.34–13.25) across eight studies. High and significant heterogeneity was found according to the *I*^2^ index, ranging from 81.6 to 92.3% (*p* < 0.001).

Nineteen studies reported a pooled mean duration of hospitalization of 12.97 days (95% CI 11.69–14.26) with significantly high heterogeneity (*I*^2^ = 97.5%, *p* < 0.001). The mean time for SARS-CoV-2 RNA in nasopharyngeal/throat swabs to become undetectable was 11.43 days (95% CI 10.1–12.77) across 13 studies. Positive fecal samples for SARS-CoV-2 were reported in 75% (95% CI 52–93%) of cases across five studies, with moderate heterogeneity (*I*^2^ = 51.56%, *p* = 0.08).

Eleven studies reported the comorbidity of pediatric COVID-19 patients. A total of 170 cases (27%, 95% CI 13–44%) had at least one comorbidity, including gastrointestinal disease (12%, 95% CI 0–35%), cardiovascular disease (6%, 95% CI 4–9%), immunosuppression/malignancy (4%, 95% CI 1–0%), and neurological disease (3%, 95% CI 1–7%). More information is given in Table 1.

**Table 1:** The demographics and epidemiological characteristics of children with COVID-19 analyzed by meta-analysis.

**Variable**	**Number of studies**	**Mean/prevalence**	**95% CI**	***n***	**Heterogeneity tests**			
					***Q***	***I*^**2**^**	***t*^**2**^**	***p***
Age (years)	49	6.48	5.20–7.75	4594	10,902.45	99.6	19.42	<0.001
<1 (%)	30	19.0	15.0–22.0	1078	109.63	73.55	0.02	<0.001
1–5 (%)	36	34.0	21.0–48.0	3116	2758.53	98.73	0.58	<0.001
≥6 (%)	37	57.0	52.0–62.0	2412	144.62	75.11	0.04	<0.001
Male (%)	54	57	55–59	3883	61.46	13.77	0.00	0.20
**Epidemiologic history (%)**								
Family cluster	39	90	85–94	970	136.86	72.23	0.09	<0.001
Endemic area exposure	34	65	49–80	897	958.11	96.56	0.72	<0.001
Contact confirmed or suspected cases	24	82	74–89	597	108.56	78.81	0.13	<0.001
Other	11	23	5–47	573	486.84	97.95	0.63	<0.001
**Epidemiological data**								
Time between exposure and symptom onset (days)	8	9.57	7.70–11.44	101	44.25	84.2	5.759	<0.001
Time between symptom onset and diagnosis (days)	15	3.07	2.54–3.60	941	182.95	92.3	0.670	<0.001
Time between exposure and diagnosis (days)	8	10.8	8.34–13.25	86	38.09	81.6	9.825	<0.001
Duration of hospitalization (days)	19	12.97	11.69–14.26	347	733.30	97.5	7.014	<0.001
Duration of virus shedding in respiratory swabs (days)	13	11.43	10.1–12.77	173	48.5	75.3	3.802	<0.001
Detection of SARS-CoV-2 RNA in stool (%)	5	75.0	52.0–93.0	41	8.26	51.56	0.12	0.08
Comorbidities (%)	11	27	13–44	170	168.2	94.05	0.27	<0.001
Cardiovascular disease	5	6	4–9	33	1.29	0.00	0.00	0.86
Immunosuppression/malignancy	7	4	1–10	28	28.03	78.6	0.05	<0.001
Gastrointestinal disease	4	12	0–35	7	39.58	92.42	0.24	<0.001
Neurological disease	4	3	1–7	13	1.71	0.00	0.00	0.63

*Q, Cochran's Q statistic for heterogeneity; I^2^, I^2^ Index for the degree of heterogeneity; t^2^, tau-squared measure of heterogeneity*.

### Clinical Characteristics

A total of 47 studies reported the symptoms of children with COVID-19, which were defined as asymptomatic infection, mild, moderate, severe, or critical on the basis of the clinical features, laboratory testing, and radiographic chest imaging. A total of 43% (95% CI 36–51%) of children across 44 studies were mild, and 52% (95% CI 42–62%) across 29 studies were moderate. Almost 23% (95% CI 15–31%) of children across 31 studies showed no specific symptoms initially. A total of 6% (95% CI 3–11%) across 10 studies were severe cases and 4% (95% CI 1–8%) were critical cases. The proportions of severe and/or critical cases were 7% (95% CI 6–8%), and 3% (95% CI 3–4%) for age groups of <5 years and more than 5 years, respectively (Supplementary Figure 1). The most prevalent clinical symptoms were fever (47%, 95% CI 41–53%), cough (42%, 95% CI 36–48%), fever and cough (30%, 95% CI 17–44%), upper respiratory tract infections (28%, 95% CI 13–45%), increased sputum production (17%, 95% CI 8–28%), dyspnea (14%, 95% CI 4–28%), and nasal congestion (14%, 95% CI 7–22%). Further analysis showed that 8% (95% CI 6–11%) of the children presented with fever higher than 39°C, 22% (95% CI 15–29%) with fever 38.01–39°C, and 20% (95% CI 12–30%) with fever lower than 38.01°C. Less frequent symptoms were sore throat (12%), sneezing (9%), nausea/vomiting (9%), fatigue (9%), wheezing (8%), shortness of breath (7%), headache/dizziness (7%), rhinorrhea (7%), diarrhea (7%), constipation (6%), anorexia (5%), and abdominal pain (4%) (Table 2).

**Table 2:** Clinical characteristics of children with COVID-19 analyzed by meta-analysis.

**Variable**	**Number of studies**	**Mean/prevalence**	**95% CI**	***N***	**Heterogeneity tests**			
					***Q***	***I*^**2**^**	***t*^**2**^**	***p***
**Severity of Illness (%)**								
Asymptomatic	31	23	15–31	495	560.47	94.65	0.17	<0.001
Mild	44	43	36–51	2185	577.61	92.56	0.16	<0.001
Moderate	29	52	42–62	1584	621.36	95.49	0.21	<0.001
Severe	10	6	3–11	164	63.19	85.76	0.03	<0.001
Critical	10	4	1–8	54	101.74	91.15	0.05	<0.001
**Clinical manifestations (%)**								
Fever	50	47	41–53	772	190.10	74.22	0.09	<0.001
37.3–38.01°C	15	20	12–30	80	50.84	72.46	0.09	<0.001
38.01–39°C	9	22	15–29	101	16.14	50.43	0.02	0.04
>39°C	12	8	6–11	47	4.01	0.00	0.00	0.97
Cough	44	42	36–48	661	157.27	72.66	0.08	<0.001
Fever and cough	5	30	17–44	53	8.74	54.24	0.05	0.07
Shortness of breath	4	7	0–19	48	40.35	92.57	0.10	<0.001
Headache/dizziness	12	7	1–15	108	142.29	92.27	0.16	<0.001
Nasal congestion	9	14	7–22	17	5.08	0.00	0.00	0.75
Sneezing	4	9	1–21	17	4.11	27.03	0.03	0.25
Rhinorrhea	18	7	5–10	54	17.83	4.63	0.00	0.40
Sore throat	22	12	5–21	185	204.12	89.71	0.21	<0.001
Sputum	11	17	8–28	109	48.06	79.19	0.11	<0.001
Dyspnea	11	14	4–28	35	68.69	85.44	0.22	<0.001
Upper airway infections	6	28	13–45	109	50.82	90.16	0.15	<0.001
Wheezing	4	8	1–17	18	7.63	60.66	0.04	0.05
Diarrhea	21	7	4–11	89	49.98	59.98	0.03	<0.001
Constipation	3	6	2–10	15	0.83	0.00	0.00	0.66
Nausea/vomiting	17	9	5–13	82	46.8	65.81	0.04	<0.001
Fatigue	13	9	5–13	40	14.11	14.95	0.01	0.29
Anorexia	5	5	0–15	12	15.71	74.53	0.07	<0.001
Abdominal pain	6	4	1–8	25	12.91	61.26	0.02	0.02

*Q, Cochran's Q statistic for heterogeneity; I^2^, I^2^ Index for the degree of heterogeneity; t^2^, tau-squared measure of heterogeneity*.

There were 23 laboratory variables analyzed in pediatric COVID-19 patients. The most common laboratory abnormalities were increases in serum creatinine kinase-MB (CK-MB) (44%, 95% CI 30–59%), procalcitonin (36%, 95% CI 17–57%), lactate dehydrogenase (LDH) (35%, 95% CI 25–47%), and neutropenia (34%, 95% CI 18–52%) followed by lymphocytosis (26%), increased C-reactive protein (23%), leukopenia (20%), and increased anti-inflammatory IL-10 (21%). A total of 17 studies reported on 255 children with COVID-19 who had concurrent infections. A total of 29% (95% CI 19–40%) of pediatric patients had a coinfection, and mycoplasma was the most common coinfection (17%, 95% CI 11–24%) followed by influenza A (7%, 95% CI 2–15%), influenza B (4%, 95% CI 1–10%), respiratory syncytial virus (2%), adenovirus (2%), and Epstein–Barr virus (3%) (Table 3).

**Table 3:** Laboratory findings of children with COVID-19 analyzed by meta-analysis.

**Variable**	**Number of studies**	**Mean/prevalence**	**95% CI**	***N***	**Heterogeneity tests**			
					***Q***	***I*^**2**^**	***t*^**2**^**	***p***
**Laboratory findings (%)**								
Leukopenia	22	20	13–27	109	80.39	73.88	0.11	<0.001
Leukocytosis	19	14	9–21	79	41.15	56.26	0.05	<0.001
Lymphopenia	22	18	10–28	131	134.7	84.41	0.20	<0.001
Lymphocytosis	21	26	16–38	147	123.99	83.87	0.21	<0.001
Neutrophilia	6	17	9–26	47	8.54	41.44	0.02	0.13
Neutropenia	12	34	18–52	82	70.27	84.35	0.25	<0.001
Decreased hemoglobin	4	17	4–33	24	58.41	7.21	0.07	0.07
Decreased albumin	4	15	1–38	35	21.84	86.27	0.20	<0.001
High platelet	7	12	7–18	24	3.30	0.00	0.00	0.77
High C-reactive protein	23	23	16–30	184	81.26	72.93	0.08	<0.001
High CK-MB	10	44	30–59	123	42.87	79.01	0.13	<0.001
High D-dimer	10	17	10–25	29	11.43	21.23	0.02	0.25
High procalcitonin level	11	36	17–57	135	130.68	92.35	0.38	<0.001
High ALT	16	13	9–18	59	18.43	18.61	0.01	0.24
High AST	14	19	11–28	57	28.18	53.87	0.06	0.01
High LDH	21	35	25–47	152	86.02	76.75	0.16	<0.001
High creatine kinase	7	18	6–34	19	17.99	66.64	0.12	0.01
High creatinine	3	4	0–16	5	5.59	64.22	0.06	0.06
High bilirubin	3	6	0–18	8	5.63	64.49	0.06	0.06
High ESR	10	19	10–29	35	20.50	56.10	0.06	0.02
High PT	4	8	0–24	7	11.51	73.94	0.12	0.01
High IL-6	6	12	4–22	33	19.39	74.21	0.06	<0.001
High IL-10	4	21	10–34	61	13.26	77.37	0.05	<0.001
Coinfection (%)	17	29	19–40	255	123.19	87.01	0.15	<0.001
Influenza A	4	7	2–15	6	0.70	0	0	0.87
Influenza B	9	4	1–10	20	32.93	75.71	0.06	<0.001
Mycoplasma	13	17	11–24	109	46.21	74.03	0.06	<0.001
Respiratory syncytial virus	9	2	0–6	16	11.32	29.34	0.01	0.18
Epstein–Barr virus	4	3	1–6	8	0.95	0	0	0.81
Adenovirus	6	2	0–4	7	1	0	0	0.96

*Q, Cochran's Q statistic for heterogeneity; I^2^, I^2^ Index for the degree of heterogeneity; t^2^, tau-squared measure of heterogeneity; CK-MB, creatine kinase-MB; ALT, alanine aminotransferase; AST, aspartate aminotransferase; LDH, lactate dehydrogenase; PT, prothrombin time; ESR, erythrocyte sedimentation rate; IL-6, interleukin-6; IL-10, interleukin-10*.

Initial chest radiological imaging in 38 pediatric COVID-19 studies is presented in Table 4. Ground glass opacities (33%, 95% CI 26–40%) alone or combined with consolidation (44%, 95% CI 20–69%) were the most common radiographic features. Bilateral pneumonia was seen in 40% (95% CI 31–50%) of cases, 30% (95% CI 25–35%) had unilateral pneumonia, and 35% (95% CI 5–72%) had subpleural lesions. Pure pulmonary consolidation occurred in 10% (95% CI 5–16%) of cases. Severe image findings of white lung and pleural effusion were reported in four and six studies at rates of 2% (95% CI 0–6%) and 1% (95% CI 0–6%), respectively. Of note, 26% (95% CI 18–35%) of cases showed no obvious radiographic abnormalities on admission.

**Table 4:** Image findings, treatments, and outcomes of children with COVID-19 analyzed by meta-analysis.

**Variable**	**Number of studies**	**Mean/prevalence**	**95% CI**	***N***	**Heterogeneity tests**			
					***Q***	***I*^**2**^**	***t*^**2**^**	***p***
**Image findings (%)**								
Normal	45	26	18–35	413	588.99	92.53	0.29	<0.001
Unilateral pneumonia	28	30	25–35	237	45.04	40.05	0.02	0.02
Bilateral pneumonia	25	40	31–50	234	116.13	79.33	0.15	<0.001
Ground-glass opacity	38	33	26–40	318	157.54	76.51	0.13	<0.001
Pulmonary consolidation	13	10	5–16	29	19.66	38.95	0.03	0.07
Ground-glass opacities and consolidation	5	44	20–69	44	21.70	81.56	0.25	<0.001
Pleural effusion	6	1	0–6	7	8.26	39.50	0.02	0.14
Subpleural lesions	7	35	5–72	46	159.39	96.24	0.80	<0.001
White lung	4	2	0–6	5	3.46	13.24	0.01	0.33
**Treatment (%)**								
Oxygen therapy	11	22	10–38	45	61.58	83.76	0.21	<0.001
Mechanical ventilation	6	9	1–23	26	36.92	86.46	0.17	<0.001
Interferon-alpha	18	81	64–95	254	187.34	90.93	0.53	<0.001
Ribavirin	12	57	26–86	158	216.91	94.93	0.94	<0.001
Oseltamivir	5	33	7–67	33	42.38	90.56	0.46	<0.001
Lopinavir/ritonavir	9	54	31–76	82	70.50	88.65	0.40	<0.001
Glucocorticoids	11	13	5–24	30	44.36	77.45	0.13	<0.001
Antibiotics	17	37	21–53	197	166.50	90.39	0.36	<0.001
Immunoglobulin	8	15	3–32	21	44.13	84.14	0.19	<0.001
Traditional Chinese medicine	6	31	14–50	36	17.15	70.84	0.12	<0.001
ICU (%)	7	7	1–14	34	26.97	77.75	0.06	<0.001
**Outcomes (%)**								
Discharged	33	79	68–88	649	290.79	89.00	0.32	<0.001
Death	3	1	0–2	6	4.68	57.27	0.01	0.1

*Q, Cochran's Q statistic for heterogeneity; I^2^, I^2^ Index for the degree of heterogeneity; t^2^, tau-squared measure of heterogeneity*.

### Treatments and Outcomes

**Figure 2:**
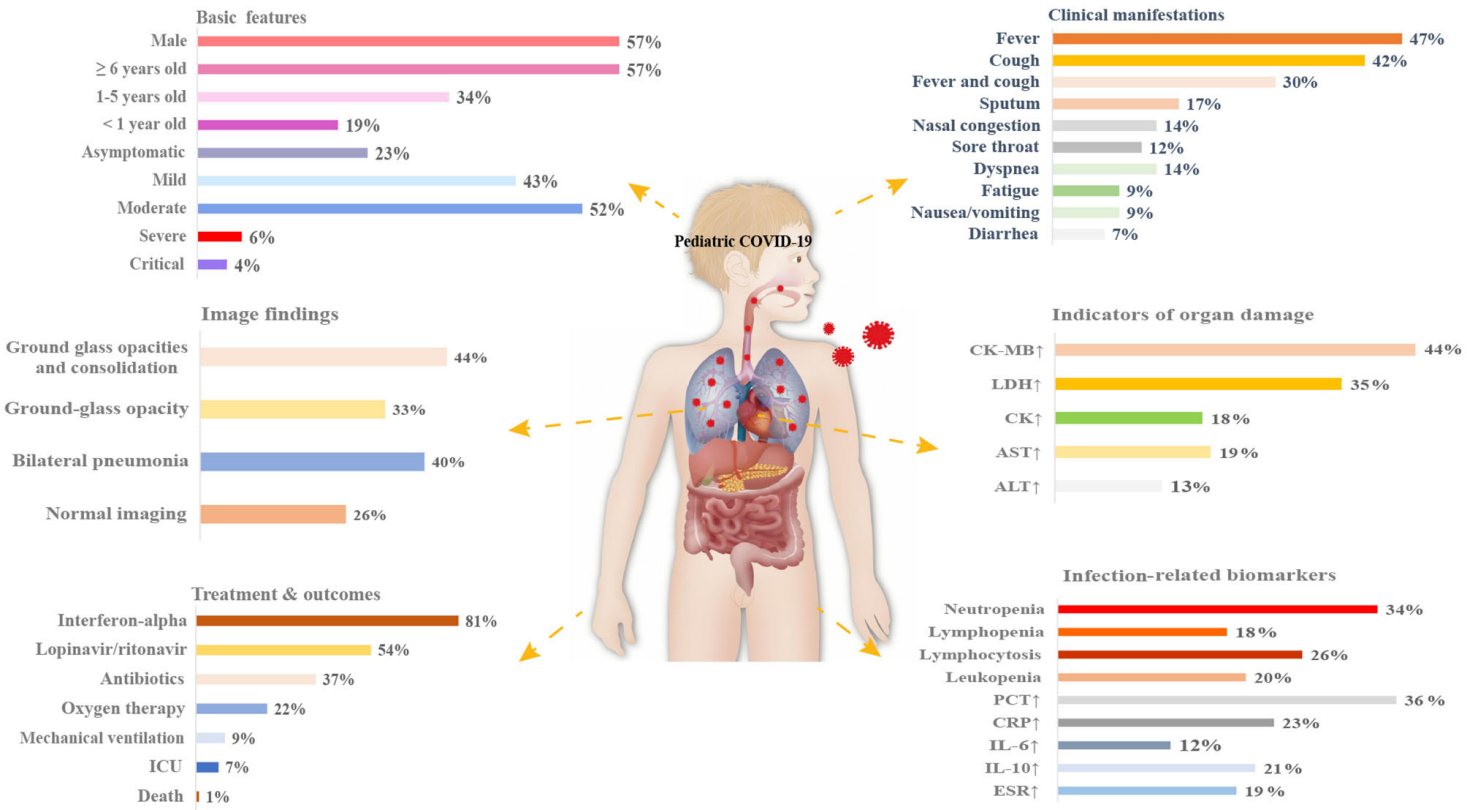
Schematic diagram depicting the presenting characteristics, treatments, and outcomes of pediatric COVID-19 patients. CK, creatine kinase; LDH, lactate dehydrogenase; ALT, alanine aminotransferase; AST, aspartate aminotransferase; PCT, procalcitonin; CRP, C-reactive protein; IL-6, interleukin-6; IL-10, interleukin-10; ESR, erythrocyte sedimentation rate; ICU, intensive care unit.

Oxygen therapy was required in 22% (95% CI 10–38%) of patients across 11 studies, and 9% (95% CI 1–23%) across six studies required mechanical ventilation. Eighteen articles reported administration of antiviral drugs such as interferon-alpha to 254 patients (81%, 95% CI 64–95%). Other antiviral drugs included ribavirin (57%, 95% CI 26–86%), lopinavir/ritonavir (54%, 95% CI 31–76%), and oseltamivir (33%, 95% CI 7–67%). In addition, both antibiotics (37%, 95% CI 21–53%) and traditional Chinese medicine (31%, 95% CI 14–50%) were used. Another 15 and 13% were treated with immunoglobulin and glucocorticoids, respectively. There were 34 patients across seven studies transferred to an ICU (7%, 95% CI 1–14%), and three studies reported an overall fatality rate of 1% (95% CI 0–2%) (Table 4). An overview of the presenting characteristics, laboratory and radiological characteristics, treatments, and outcomes of pediatric patients with COVID-19 is shown in Figure 2.

### Case Reports

Forty-two case reports of 53 children with COVID-19 were included (Supplementary Table 8). The ages of the patients were 0–13 years with a mean of 3.7 years, and the male/female ratio was 1.74:1. The most reported clinical features were fever (70.9%), cough (43.6%), nasal symptoms (29.1%), diarrhea (23.6%), myalgia or fatigue (20.0%), nausea or vomiting (20.0%), shortness of breath/dyspnea (14.5%), and sputum production (10.9%). In addition, 10.9% of the cases showed no symptoms. The most common laboratory abnormalities were increases in C-reactive protein (34.0%), procalcitonin (21.3%), and CK-MB (19.1%). A total of 76.6% of the cases presented with abnormal radiographic features, including bilateral pneumonia (40.4%), patchy shadowings (27.7%), and ground-glass opacities (25.5%). The majority (65.2%) of the cases were treated with interferon-alpha, 39.1% received antibiotic treatment, 17.4% received oxygen therapy, and 8.7% received assisted ventilation. In addition, 15.2% were treated with immunoglobulin and traditional Chinese medicine (Table 5).

**Table 5:** Summary of the case report findings on COVID-19, 2020.

**Variable**	***n***	%*
Age (months) (mean, *SD*) (*n* = 53)	44.4, 48.8	44.8
Sex (male/female) (*n* = 52)	33	64.5
**Clinical features**		
Fever	39/53	73.4
Cough	23/53	43.4
Shortness of breath/dyspnea	8/53	15.1
Sore throat	2/53	3.8
Diarrhea	13/53	24.5
Nasal symptoms	14/53	26.4
Myalgia or fatigue	11/53	20.8
Sputum production	6/53	11.3
Nausea or vomiting	11/53	20.8
No symptoms	5/53	9.4
**Laboratory findings**		
Normal	8/47	17.0
Leukopenia	6/47	12.8
Leukocytosis	7/47	14.9
Lymphopenia	6/47	12.8
High AST	6/47	12.8
High ALT	2/47	4.3
High LDH	8/47	17.0
High C-reactive protein	16/47	34.0
High procalcitonin	10/47	21.3
High creatinine	2/47	4.3
High creatine kinase	6/47	12.8
High CK-MB	9/47	19.1
High bilirubin	4/47	8.5
Decreased albumin	2/47	4.3
**Images**		
Normal	11/45	24.4
Abnormal	34/45	75.6
Unilateral pneumonia	10/45	22.2
Bilateral pneumonia	19/45	42.2
Ground-glass opacity	12/45	26.7
Pulmonary consolidation	9/45	20.0
Patchy shadowings	13/45	28.9
**Treatment**		
Symptomatic treatment	9/44	20.5
Oxygen supply	7/44	15.9
Noninvasive/mechanical ventilation	4/44	9.1
Oseltamivir	9/44	20.5
Ribavirin	5/44	11.4
Lopinavir/litonavir/ribavirin	4/44	9.1
Interferon-alpha	30/44	68.2
Glucocorticoids	5/44	11.4
Immunoglobulin	7/44	15.9
Antibiotics	18/44	40.9
Traditional Chinese medicine	7/44	15.9

*ALT, alanine aminotransferase; AST, aspartate aminotransferase; LDH, lactate dehydrogenase; CK-MB, creatine kinase-MB*.

* *Expressed in absolute number and percentage in relation to the total of cases described (n = 53)*.

## Discussion

The risk of SARS-CoV-2 transmission through children should not be ignored. Given the low rate of infection in children, children are thought to be less likely to become infected when exposed to the virus compared with adults. However, 90% of all the pediatric cases in our analysis were infected through close contact with family members with COVID-19, which was the main route of transmission when schools and daycares were closed, indicating that children are as vulnerable as adults to SARS-CoV-2 infection. Furthermore, the shedding of SARS-CoV-2 in the upper respiratory tract of pediatric patients lasted 11.43 days, which is comparable with adults (13). A Chicago study showed that children with COVID-19 younger than 5 years old had 10–100 times viral load compared with children more than 5 years old and adults with COVID-19 (14). A series of 228 diagnosed SARS-CoV-2 infections in France indicated that the incidence of COVID-19 infection increased by 7.4-fold in children between 1 and 5 years old (15). According to Dong et al. (16), severe illness is generally seen in patients younger than 1 year of age. In our analysis, we found that children <5 years had more severe symptoms, but further studies are needed to confirm our observations. Therefore, their role in transmission might be underestimated. Recent studies have inferred that live viruses and viral nucleic acids can be detected in the stool of patients with COVID-19, and the RNA load remains steadily high even when it declines in nasopharyngeal swab specimens (17–19). This was confirmed by our analysis, indicating the potential infectiousness of feces among children. Furthermore, 23% of children were asymptomatic in this analysis, which was significantly higher than that reported by the Chinese CDC (3), thus making it difficult to identify children as the index patients. In addition, 26% of pediatric cases showed normal chest radiological imaging on admission, which also makes it difficult to identify the suspected cases. Consistent with the findings of Guo et al. (20), our results revealed that the pooled mean incubation period in children was 9.57 days, which was longer than adults (4 days) (21). This might be explained by children being less likely to be tested for coronavirus infection due to the proxy reports of symptoms in younger children, especially at pre-school age. On the other hand, children might not get tested in a timely manner in some countries due to a lack of local resources and policies on testing and contact tracing. This means that there is a higher risk of SARS-CoV-2 transmission through children, and thus extensive preventive strategies for children are recommended to control the spread of SARS-CoV-2 among household contacts and in schools.

When compared with adults, most pediatric patients presented with mild or moderate clinical syndromes, and only a few were admitted to the ICU, which was much less than that of adults. As reported by Tang et al. (22), almost 26–32% of adult patients were committed to the ICU. Fever and cough were the dominant symptoms in pediatric patients, of which the frequencies were lower compared with adults (55.49–78.49%). The duration of hospital stay was also shorter than that of adults (19 days) (23). Similar with the findings of Zhu et al. (24), myocardial enzyme spectrum abnormalities (increased CK-MB and LDH in 44 and 35% of cases) were more common in pediatric patients, which might be caused by different degrees of myocardial cell damage caused by infection. However, infection-related biomarkers (IL-6 and IL-10) were less frequently observed, indicating that systemic inflammation was weaker in pediatric patients, which might be related to mild to moderate cases being more common in children. However, we must take caution when interpreting these outcomes due to substantial heterogeneity that might have affected the overall quality of the evidence. The heterogeneity was mainly associated with dissimilarities of the included studies in terms of sample size, design, and location. The case reports showed a higher proportion of males, and recent studies have suggested that being male is also a factor in the epidemiology of COVID-19 due to the biological differences in the immune systems between males and females (25). However, further epidemiological investigations are needed to prove that male children are more susceptible. In addition, serum inflammatory markers, specifically C-reactive protein, procalcitonin, and CK-MB, were abnormal in children with COVID-19. Although Bikash et al. (26) highlighted the importance of C-reactive protein as a possible biomarker for mortality from COVID-19 infection, its impact on disease severity in the pediatric population is unknown. The discrepancy between meta-analyses and case reports was attributed to small sample sizes and selection bias in the case reports.

As for the diagnosis of COVID-19 in pediatric patients, these were confirmed by laboratory tests of samples that were taken from upper nasopharyngeal swabs. Although nucleic acid detection is the gold standard, it is easy to have a false negative due to the influence of many factors on the specimen (27). In addition, these clinical symptoms have no obvious specificity compared with other cases of pneumonia. It is likely that these changes in blood biochemical indexes are non-specific and might merely indicate an inflammatory state induced by the virus. Therefore, we can comprehensively judge the cases of COVID-19 with histories of epidemiology, clinical symptoms, myocardial enzyme spectrum, and chest radiological imaging.

Co-infection with SARS-CoV-2 and other respiratory pathogens in pediatric patients was higher than in adults (28). The most common coinfection in children was mycoplasma. A study on patients with mycoplasma pneumonia and COVID-19 pneumonia suggested that they may have similar presentations in clinical and radiographic characteristics (29). Thus, COVID-19 is easily overlooked in the presence of mycoplasma coinfection. In addition, several studies suggest that co-infection between SARS-CoV-2 and other respiratory pathogens is associated with stronger inflammation response, protracted respiratory symptoms, and increased severity (30, 31). However, more studies are needed to assess the effect of SARS-CoV-2 and influenza co-infection in terms of clinical outcomes. Given the longer detection time of SARS-CoV-2 viruses, it is important to routinely test for SARS-CoV-2 viruses among children with mycoplasma pneumonia and to treat them appropriately during the COVID-19 pandemic.

Currently, the only therapeutic recommendation for pediatric patients in China is nebulized interferon-alpha (IFN-α) and oral anti-viral drugs. IFN-α has been shown to exert a protective effect against SARS-CoV infection (32). Lopinavir/ritonavir and IFN-α therapy were used as prioritized drugs in the WHO's SOLIDARITY trial, and our results showed that most children with COVID-19 were treated with IFN-α. Antibiotics were also used frequently to defend against secondary bacterial or mycoplasma infections. One matched case–control study on the evaluation of the clinical effects of IFN-α treatment in adult patients indicated that IFN-α improved discharge rates, reduced hospitalization time, and reduced virus shedding time (33). However, well-designed large-sample randomized studies are needed for a more definitive evaluation of IFN-α treatment for pediatric patients. Hydroxychloroquine has been in widespread use for the treatment of COVID-19 in India (34), Turkey (35), and North America (36), but prolongation of QTc interval has been reported in hydroxychloroquine-treated COVID-19 patients, even those with only mild to moderate disease (37). Remdesivir, glucocorticoids, tocilizumab, and convalescent plasma have also been used as therapeutic options in severe pediatric patients, but these therapies should be used with caution because of their side effects and lack of evidence for their efficacy (38–43). Furthermore, it is noteworthy that patient selection is critical when using these novel therapies to avoid harm (40, 42).

This analysis showed clear age-related differences in clinical characteristics of COVID-19, which might be related to dissimilar immune responses to SARS-CoV-2. First, aging is associated with increased expression of the cell surface enzyme ACE2 in the nasal epithelium (44), which has been proven to bind to the SARS-CoV-2 spike protein and promote internalization of the virus into human cells. Second, age-related excessive neutrophil recruitment induces tissue injury and worsens disease, and it has been reported that aging increases mortality from influenza in mice because of increased neutrophil accumulation (45). Third, numerous studies have highlighted that lymphopenia is the most common abnormality among adult patients, in contrast with the findings in pediatric patients, and the replenishment of lymphocytes killed by the SARS-CoV-2 virus is thought to be critical for disease control and prognosis (46).

Our review has several limitations. First, all of the included studies were retrospective studies, and some studies were single-center or preprinted articles that had not been peer reviewed, so we cannot rule out the influence of the significant heterogeneity observed between studies. Second, most of the studies included in our analysis came from China, so we could not assess the race or ethnicity data. These results therefore might not be representative of high-risk groups such as African children who also have higher expressions of ACE2 (47). Third, the pandemic is still spreading and the available data were accumulated over a short period of time. Recent reports from the USA (48), Italy (49), and the UK (50) suggest a new COVID-19-related clinical syndrome called multisystem inflammatory syndrome in children (MIS-C), which is characterized by significant inflammation and other similarities to Kawasaki disease. Nevertheless, pediatric cases with MIS-C have rarely been reported in the studies from China. Further cohort or case–control studies are urgently needed to establish the causality between COVID-19 and MIS-C. Fourth, because young children have problems with describing their health status, this will inevitably result in under-detected cases in the community or in delayed presentation. Hence, the infection rates, estimated incubation period, and timing to diagnosis are limited to the studies describing symptomatic patients admitted in the hospital. In addition, given the one-child policy that has been in place for a long time in China, current Chinese family units might be smaller than those in western countries. This might have impacted the pediatric infective rates and transmission rates within family contacts. Lastly, due to limited data, we were unable to assess more detailed clinical information, subgroup analysis, and sensitivity analysis of all sources of heterogeneity that might have affected the accuracy of the results were not preformed.

## Conclusions

In summary, the presenting characteristics, comorbidities, and severity of illness of pediatric patients with COVID-19 were different, and milder, compared with adults. All ages of children can potentially transmit SARS-CoV-2, but children are less likely than adults to be symptomatic and are more susceptible to co-infection, which make diagnosis and infection source control more challenging.

## Data Availability Statement

All datasets generated for this study are included in the article/Supplementary Material.

## Author Contributions

CZ was responsible for study design, supervision, analysis, and interpretation of the data. BL and SZ were responsible for acquisition, analysis, interpretation of data, statistical analysis, and drafting of the article. RZ, XC, and YW were responsible for data searching, extraction, and synthesis. All authors contributed to the article and approved the submitted version.

## Conflict of Interest

The authors declare that the research was conducted in the absence of any commercial or financial relationships that could be construed as a potential conflict of interest.
