# Supplementary material for: Epidemiological and Clinical Characteristics of COVID-19 in Children: A Systematic Review and Meta-Analysis
Source: Front Pediatr. 2020 Nov 2;8:591132. doi: 10.3389/fped.2020.591132 (PMC7667131; doi:10.3389/fped.2020.591132)
Supplement: Supplementary file 1 [file Table_1.DOCX]

**Supplementary Table 1 The full search strategy of electronic databases**

| **Database** |  | **Date** | **Hits** |
| --- | --- | --- | --- |
| **PubMed /Medline** | ("severe acute respiratory syndrome coronavirus 2"[Supplementary Concept] OR "severe acute respiratory syndrome coronavirus 2"[All Fields] OR "sars cov 2"[All Fields] OR ("severe acute respiratory syndrome coronavirus 2"[Supplementary Concept] OR "severe acute respiratory syndrome coronavirus 2"[All Fields] OR "2019 ncov"[All Fields]) OR "coronavirus disease 2019"[Title/Abstract] OR "severe acute respiratory syndrome coronavirus 2"[Title/Abstract] OR "COVID-19"[Title/Abstract]) AND ("child"[MeSH Terms] OR "child"[All Fields] OR "children"[All Fields] OR "child s"[All Fields] OR "children s"[All Fields] OR "childrens"[All Fields] OR "childs"[All Fields] OR "children"[Title/Abstract] OR ("infant"[MeSH Terms] OR "infant"[All Fields] OR "infants"[All Fields] OR "infant s"[All Fields]) OR ("infant, newborn"[MeSH Terms] OR ("infant"[All Fields] AND "newborn"[All Fields]) OR "newborn infant"[All Fields] OR "newborn"[All Fields] OR "newborns"[All Fields] OR "newborn s"[All Fields]) OR "neonate"[Title/Abstract] OR ("adolescences"[All Fields] OR "adolescency"[All Fields] OR "adolescent"[MeSH Terms] OR "adolescent"[All Fields] OR "adolescence"[All Fields] OR "adolescents"[All Fields] OR "adolescent s"[All Fields]) OR "teenagers"[Title/Abstract] OR ("paediatrics"[All Fields] OR "pediatrics"[MeSH Terms] OR "pediatrics"[All Fields] OR "paediatric"[All Fields] OR "pediatric"[All Fields])) | May 7, 2020 | 557 |
| **Web of Science** | #1 TOPIC: " SARS-CoV-2"  #2 TOPIC: " 2019-nCoV"  #3 TOPIC: " coronavirus disease 2019"  #4 TOPIC: " severe acute respiratory syndrome coronavirus 2"  #5 TOPIC: " COVID-19”  #6 #1-5/OR  #7 TOPIC: " child"  #8 TOPIC: "children"  #9 TOPIC: "infant"  #10 TOPIC: "newborn"  #11 TOPIC: "pediatrics"  #12 TOPIC: "paediatrics"  #13 TOPIC: "adolescent"  #14 #7-13/ OR  #15 #6 AND #14 | May 7, 2020 | 307 |
| **OVID** | 1 " SARS-CoV-2"  2 " 2019-nCoV"  3 " coronavirus disease 2019"  4 " severe acute respiratory syndrome coronavirus 2"  5 " COVID-19”  6 1 OR 2 OR 3 OR 4 OR 5  7 "child"  8 "children"  9 "infant"  10 "newborn"  11 "pediatrics"  12 "paediatrics"  13 "adolescent"  14 7 OR 8 OR 9 OR 10 OR 11 OR 12 OR 13  15 6 AND 14 | May 7, 2020 | 82 |
| **Wan Fang Data** | 1 "新型冠状病毒"[主题]  2 " SARS-CoV-2"[主题]  3 " 2019-nCoV" [主题]  4 " coronavirus disease 2019"[主题]  5 " severe acute respiratory syndrome coronavirus 2"[主题]  6 " COVID-19” [主题]  7 1-6/OR  8 "儿童" [主题]  9 "婴幼儿" [主题]  10 "新生儿" [主题]  11 "青少年" [主题]  12 "少年" [主题]  13 8-12/OR  14 7 AND 13 | May 7, 2020 | 31 |
| **CNKI** | 1 "新型冠状病毒"[主题]  2 " SARS-CoV-2"[主题]  3 " 2019-nCoV" [主题]  4 " coronavirus disease 2019"[主题]  5 " severe acute respiratory syndrome coronavirus 2"[主题]  6 " COVID-19” [主题]  7 1-6/OR  8 "儿童" [主题]  9 "婴幼儿" [主题]  10 "新生儿" [主题]  11 "青少年" [主题]  12 "少年" [主题]  13 8-12/OR  14 7 AND 13 | May 7, 2020 | 104 |
| **Chinese Medical Journal full-text database** | 1 "新型冠状病毒"[主题]  2 " SARS-CoV-2"[主题]  3 " 2019-nCoV" [主题]  4 " coronavirus disease 2019"[主题]  5 " severe acute respiratory syndrome coronavirus 2"[主题]  6 " COVID-19” [主题]  7 1-6/OR  8 "儿童" [主题]  9 "婴幼儿" [主题]  10 "新生儿" [主题]  11 "青少年" [主题]  12 "少年" [主题]  13 8-12/OR  14 7 AND 13 | May 7, 2020 | 69 |
