# Supplementary material for: Epidemiological and Clinical Characteristics of COVID-19 in Children: A Systematic Review and Meta-Analysis
Source: Front Pediatr. 2020 Nov 2;8:591132. doi: 10.3389/fped.2020.591132 (PMC7667131; doi:10.3389/fped.2020.591132)
Supplement: Supplementary file 2 [file Table_2.DOCX]

**Supplementary Table 2 Characteristics of the included studies on COVID-19, 2020**

| ID | Study | Journal | Date  (MM/DD) | Country | Study type | No. | | | | | Mean age ± SD or Median age (range) | Quality score ^a^ |
| --- | --- | --- | --- | --- | --- | --- | --- | --- | --- | --- | --- | --- |
|  |  |  |  |  |  | Total cases | Age <1y | Age 1-5y | Age >6y | Sex  (male) |  |  |
| 1 | Cai et al (1) | IDSA | 02/28 | China | Case series | 10 | 2 | 2 | 6 | 4 | 7 (0.25-10.92) | 15 |
| 2 | Hu et al (2) | Life Sci | 03/03 | China | Case series | 6 | - | 1 | 5 | 3 | 9.67 ± 4.13 | 15 |
| 3 | Zhu et al (3) | Pediatr Pulm | 03/24 | China | Case series | 10 | - | 2 | 8 | 5 | 9.16 ± 4.66 | 12 |
| 4 | CDC  COVID-19 Team (4) | cdc.gov/mmwr/ | 04/10 | USA | Case series | 2572 | 398 | 2174 | - | 1408 | 11 (0-17) | 14 |
| 5 | Turner et al (5) | J Pediatr Gastr Nut R | 03/26 | Multiple | Case series | 6 | - | - | 6 | 3 | 16.6 ± 1.79 | 13 |
| 6 | Liu et al (6) | NEJM | 04/02 | China | Case series | 6 | 1 | 4 | 1 | 2 | 3 (1-7) | 13 |
| 7 | Dong et al (7) | Pediatrics | 03/16 | China | Case series | 728 | 85 | 137 | 506 | 418 | 10 (4-15) * | 13 |
| 8 | Liu et al(8) | J Comput Assist Tomogr | 03/25 | China | Case series | 5 | 1 | 2 | 2 | 4 | 6.04 ± 5.02 | 13 |
| 9 | Tagarro et al(9) | JAMA Pediatrics | 04/08 | Spain | Case series | 41 | - | - | - | 18 | 1 (0-15) | 15 |
| 10 | Su et al(10) | Emerg Microbes Infec | 03/12 | China | Case series | 9 | 2 | 5 | 2 | 3 | 3.50 (0.92-9) | 16 |
| 11 | Xu et al(11) | Nat Med | 03/02 | China | Case series | 10 | 1 | 2 | 7 | 6 | 6.63 (0.17-15.67) | 14 |
| 12 | Li et al(12) | Pediatr Radiol | 03/02 | China | Case series | 5 |  | 4 | 1 | 4 | 3.05 ± 2.07 | 13 |
| 13 | Xia et al(13) | Pediatr Pulm | 02/26 | China | Case series | 20 | 9 | 8 | 3 | 13 | 2.22 (0-14.58) | 12 |
| 14 | Liu et al(14) | J Infection | 03/02 | China | Case series | 4 | 2 | 1 | 1 | 2 | 3.77 ± 4.08 | 13 |
| 15 | Qiu et al(15) | Lancet Infect Dis | 03/25 | China | Cohort study | 36 | - | - | 26 | 23 | 8.3 ± 3.5 | 6 ^b^ |
| 16 | Zheng et a(16) | Curr Med Sci | 03/24 | China | Cross-sectional | 25 | - | - | 9 | 14 | 3 (0.25-14) | 17 ^c^ |
| 17 | Sun et al(17) | World J Pediatr | 03/02 | China | Case series | 8 | 1 | 2 | 5 | 6 | 6.81 ± 6.52 | 15 |
| 18 | Shen et al(18) | Pediatr Pulm | 03/23 | China | Case series | 9 |  | 2 | 7 | 3 | 7.56 ± 3.71 | 15 |
| 19 | Li et al(19) | Indian Pediatr | 04/04 | China | Case series | 22 | - | - | - | 12 | 8±6 | 12 |
| 20 | Li et al(20) | J Infection | 04/02 | China | Cross-sectional | 40 | - | - | -- | 23 | 5.09 ± 4.71 | 14 ^c^ |
| 21 | Han et al(21) | J Med Virol | 03/29 | China | Cross-sectional | 7 | - | - | - | 4 | 1.3 (0.2-13) | 13 ^c^ |
| 22 | Du et al(22) | Infection | 03/23 | China | Cross-sectional | 14 | - | - | - | 6 | 6.2 (0-16) | 15 ^c^ |
| 23 | Wei et al(23) | JAMA | 04/07 | China | Case series | 9 | 9 | - | - | 2 | 0.58 ± 0.55 | 16 |
| 24 | See et al(24) | Int J Infect Dis | 03/20 | Malaysia | Case series | 4 | - | - | 4 | 3 | 6.5 ± 6.42 | 12 |
| 25 | Lu et al(25) | NEJM | 03/18 | China | Case series | 171 | 31 | 40 | 100 | 104 | 6.7 (0-15) | 17 |
| 26 | Ma et al(26) | J Microbiol | 03/11 | China | Case series | 6 | 1 | 3 | 2 | 2 | 4.65 ± 3.18 | 13 |
| 27 | Wang et al(27) | J Aller Clin  Immunol Pract | 04/14 | China | Case series | 2143 | 379 | 493 | 1271 | 1213 | - | 13 |
| 28 | Tang et al(28) | MedRxiv | 03/10 | China | Case series | 26 | - | 13 | 13 | 17 | 6.9 ± 0.7 | 14 |
| 29 | Peng et al(29) | MedRxiv | 04/10 | China | Case series | 35 | - | - | - | 15 | 7.2 (3.9-11.4) | 13 |
| 30 | Wu et al(30) | MedRxiv | 03/26 | China | Case series | 74 | 16 | - | 58 | 44 | 6 (0.10-15.08) | 18 |
| 31 | Liu et al(31) | Lancet Infect Dis |  | China | Case series | 248 | 45 | 58 | 145 | 153 | 7.18 (2.24-10.89) | 15 |
| 32 | Yu et al(32) | MedRxiv | 03/18 | China | Case series | 82 | 25 | 22 | 35 | 51 | 0-16 | 13 |
| 33 | Zhang et al(33) | MedRxiv | 03/16 | China | Case series | 34 | - | - | - | 14 | 2.75 (0.08-12) | 16 |
| 34 | Tan et al(34) | J Clin Virol | 04/03 | China | Case series | 10 | - | 3 | 7 | 3 | 8.79 (1.08-12.08) | 13 |
| 35 | Xu et al(35) | MedRxiv | 04/23 | China | Case series | 32 | - | 10 | 22 | 17 | 8.7 ± 4.7 | 18 |
| 36 | Shekerdemian et al(36) | JAMA Pediatrics | 05/07 | USA | Cross-sectional | 48 | 8 | 6 | 34 | 25 | 13 (4.2-16.6) * | 17 ^c^ |
| 37 | Liu et al(37) | Chin J Nosocomiol | 03/18 | China | Case series | 91 | 23 | 38 | 30 | 56 | 4 (0-15) | 12 |
| 38 | Ji et al(38) | J Pediat Pharmacy | 03/09 | China | Case series | 4 | 2 | 1 | 1 | 3 | 0.87 (0.75-10) | 13 |
| 39 | Wang et al(39) | Chin J Pediatr | 02/26 | China | Case series | 31 | - | - | - | 15 | 7.08 (0.5-17) ^#^ | 15 |
| 40 | Zhou et al(40) | Chin J Contemp Pediatr | 02/27 | China | Case series | 9 | 3 | 6 | - | 4 | 1 (0.58-3) | 11 |
| 41 | Ma et al(41) | Chin J Contemp Pediatr | 03/16 | China | Case series | 115 | 14 | 36 | 65 | 73 | 0.14-15 | 11 |
| 42 | Tan et al(42) | Chin J Contemp Pediatr | 03/30 | China | Case series | 13 | - | 4 | 9 | 4 | 8 (1-17) | 12 |
| 43 | Feng et al(43) | Chin J Pediatr | 02/10 | China | Case series | 15 | - | 4 | 11 | 5 | 7 (4-14) | 12 |
| 44 | Yang et al(44) | J ShanDong Uni  (Health Sci) | 03/09 | China | Case series | 10 | 2 | 5 | 3 | 3 | 5.08 ± 4.17 | 13 |
| 45 | Jiang et al(45) | Chin Pediatr Emer Med | 02/25 | China | Case series | 6 | 1 | 2 | 3 | 5 | 7.42 ± 5.55 | 11 |
| 46 | Zhang et al(46) | J ShanDong Uni  (Health Sci) | 02/19 | China | Case series | 10 | 2 | 3 | 5 | 3 | 4 (0.92-14) | 11 |
| 47 | Wu et al(47) | Chin J Contemp Pediatr | 04/03 | China | Case series | 23 | - | - | 6 | 9 | 5.58 (0.25-17.67) | 15 |
| 48 | Li et al(48) | Radiol Practice | 03/04 | China | Case series | 30 | 4 | 14 | 16 | 18 | 6 (0-14) | 11 |
| 49 | Xiong et al(49) | Chongqing Med | 04/03 | China | Case series | 6 | 1 | 2 | 3 | 4 | 0.67-13 | 12 |
| 50 | Zheng et al(50) | J Cent South Univ | 03/05 | China | Case series | 9 | 1 | 5 | 6 | 4 | 6.48 ± 4.33 | 13 |
| 51 | Ma et al(51) | Chin J Radiology | 02/06 | China | Case series | 22 | 4 | 10 | 8 | 12 | 5.54 ± 3.46 | 11 |
| 52 | Chen et al(52) | Chin J Contemp Pediatr | 04/17 | China | Case series | 20 | - | - | - | 7 | 7.8 ± 5.4 | 12 |
| 53 | Yang et al(53) | J Guangzhou Uni Tradit Chin Med | 03/19 | China | Case series | 11 | - | - | - | 8 | 8.17 (1.17-15.58) | 11 |
| 54 | Feng et al(54) | Chin J Pediatr | 02/28 | China | Case series | 5 | 5 | - | - | 3 | - | 14 |

* Represents the median (interquartile range); ^#^ Represents the mean (range). ^a^ Quality score ranged from 0 to 20 based on the Appraisal Tool for Case Series, IHE; ^b^ Quality score ranged from 0 to 20 based on the Appraisal Tool for Cross-Sectional Studies, AXIS. ^c^ Quality score ranged from 0 to 9 based on the Appraisal Tool for Cohort Studies, NOS.

**References**

1. Cai J, Xu J, Lin D, Yang Z, Xu L, Qu Z, et al. A Case Series of children with 2019 novel coronavirus infection: clinical and epidemiological features. Clin Infect Dis [Preprint] (2020).

Available at: https://academic.oup.com/cid/article/doi/10.1093/cid/ciaa198/5766430# (Accessed February 28, 2020).

2. Hu Z, Song C, Xu C, Jin G, Chen Y, Xu X, et al. Clinical characteristics of 24 asymptomatic infections with COVID-19 screened among close contacts in Nanjing, China. Sci China Life Sci (2020) 63(5):706-711. doi: 10.1007/s11427-020-1661-4

3. Zhu L, Wang J, Huang R, Liu LG, Zhao HY, Wu C, et al. Clinical characteristics of a case series of children with coronavirus disease 2019. Pediatric Pulmonology (2020) 55(6):1430-2. doi: 10.1002/ppul.24767

4. Team CC-R. Coronavirus disease 2019 in children - United States, February 12-April 2, 2020. MMWR Morb Mortal Wkly Rep (2020) 69(14):422-6. doi: 10.15585/mmwr.mm6914e4

5. Turner D, Huang Y, Martín-de-Carpi J, Aloi M, Focht G, Kang B, et al. COVID-19 and paediatric inflammatory bowel diseases: global experience and provisional guidance (March 2020) from the Paediatric IBD Porto group of ESPGHAN. J Pediatr Gastr Nutr (2020) 70(6):727-733 doi: 10.1097/mpg.0000000000002729

6. Liu WY, Zhang Q, Chen JB, Xiang R, Song HJ, Shu SN, et al. Detection of covid-19 in children in early January 2020 in Wuhan, China. N Engl J Med (2020) 382(14):1370-1. doi: 10.1056/NEJMc2003717

7. Dong Y, Mo X, Hu Y, Qi X, Jiang F, Jiang Z, et al. Epidemiology of COVID-19 among children in China. Pediatrics (2020) 145(6):e20200702. doi: 10.1542/peds.2020-0702

8. Liu M, Song Z, Xiao K. High-Resolution computed tomography Manifestations of 5 pediatric patients with 2019 novel coronavirus. J Comput Assist Tomo (2020) 44(3):311-313.

9. Tagarro A, Epalza C, Santos M, Sanz-Santaeufemia FJ, Otheo E, Moraleda C, et al. Screening and severity of coronavirus disease 2019 (COVID-19) in children in Madrid, Spain. JAMA Pediatr (2020) e201346. doi: 10.1001/jamapediatrics.2020.1346

10. Su L, Ma X, Yu HF, Zhang ZH, Bian PF, Han YL, et al. The different clinical characteristics of corona virus disease cases between children and their families in China - the character of children with COVID-19. Emerg Microbes Infec (2020) 9(1):707-13. doi: 10.1080/22221751.2020.1744483

11. Xu Y, Li XF, Zhu B, Liang HY, Fang CX, Gong Y, et al. Characteristics of pediatric SARS-CoV-2 infection and potential evidence for persistent fecal viral shedding. Nat Med (2020) 26(4):502-505. doi: 10.1038/s41591-020-0817-4

12. Li W, Cui HQ, Li KW, Fang YJ, Li SL. Chest computed tomography in children with COVID-19 respiratory infection. Pediatr Radiol (2020) 50(6):796-799. doi: 10.1007/s00247-020-04656-7

13. Xia W, Shao JB, Guo Y, Peng XH, Li Z, Hu DY. Clinical and CT features in pediatric patients with COVID-19 infection: Different points from adults. Pediatr Pulmonol (2020) 55(5):1169-74. doi: 10.1002/ppul.24718

14. Liu HH, Liu F, Li JN, Zhang TT, Wang DB, Lan WS. Clinical and CT imaging features of the COVID-19 pneumonia: Focus on pregnant women and children. J Infect (2020) 80(5):E7-E13. doi: 10.1016/j.jinf.2020.03.007

15. Qiu H, Wu J, Hong L, Luo Y, Song Q, Chen D. Clinical and epidemiological features of 36 children with coronavirus disease 2019 (COVID-19) in Zhejiang, China: an observational cohort study. Lancet Infect Dis (2020) 20(6):689-696. doi: 10.1016/s1473-3099(20)30198-5

16. Zheng F, Liao C, Fan QH, Chen HB, Zhao XG, Xie ZG, et al. Clinical Characteristics of Children with Coronavirus Disease 2019 in Hubei, China. Curr Med Sci (2020) 40(2):275-280. doi: 10.1007/s11596-020-2172-6

17. Sun D, Li H, Lu XX, Xiao H, Ren J, Zhang FR, et al. Clinical features of severe pediatric patients with coronavirus disease 2019 in Wuhan: a single center's observational study. World J Pediatr (2020) 19:1-9. doi: 10.1007/s12519-020-00354-4

18. Shen Q, Guo W, Guo T, Li J, He W, Ni S, et al. Novel coronavirus infection in children outside of Wuhan, China. Pediatr Pulmonol (2020) 55(6):1424-1429. doi: 10.1002/ppul.24762

19. Li B, Shen J, Li L, Yu C. Radiographic and Clinical Features of Children with 2019 Novel Coronavirus (COVID-19) Pneumonia. Indian Pediatr (2020) 57(5):423-426.

20. Li H, Chen K, Liu M, Xu H, Xu Q. The profile of peripheral blood lymphocyte subsets and serum cytokines in children with 2019 novel coronavirus pneumonia. J Infect (2020) 81(1):115-120.

21. Han YN, Feng ZW, Sun LN, Ren XX, Wang H, Xue YM, et al. A comparative-descriptive analysis of clinical characteristics in 2019-coronavirus-infected children and adults. J Med Virol (2020) 1– 7. doi: 10.1002/jmv.25835

22. Du W, Yu J, Wang H, Zhang X, Zhang S, Li Q, et al. Clinical characteristics of COVID-19 in children compared with adults in Shandong Province, China Infection (2020) 48(3): 445-452. doi: 10.1007/s15010-020-01427-2

23. Wei M, Yuan JP, Liu Y, Fu T, Yu X, Zhang ZJ. Novel coronavirus infection in hospitalized infants under 1 year of age in China. JAMA (2020) 323(13):1313-4. doi: 10.1001/jama.2020.213110.1001/jama.2020.2131

24. See KC, Liew SM, Ng DCE, Chew EL, Khoo EM, Sam CH, et al. COVID-19: Four paediatric cases in Malaysia. Int J Infect Dis (2020) 94:125-7.

25. Lu XX, Zhang LQ, Du H, Zhang JJ, Li YY, Qu JY, et al. SARS-CoV-2 infection in children. N Engl J Med (2020) 382(17):1663-5. doi: 10.1056/NEJMc2005073

26. Ma X, Su L, Zhang Y, Zhang X, Gai Z, Zhang Z. Do children need a longer time to shed SARS-CoV-2 in stool than adults? J Microbiol Immunol Infect (2020) 53(3):373-376. doi: 10.1016/j.jmii.2020.03.010

27. Wang E, Brar K. COVID-19 in children: an epidemiology study from China. J Allergy Clin Immunol Pract (2020)8(6): 2118–2120.

28. Tang A, Xu W, shen m, Chen P, Li G, Liu Y, et al. A retrospective study of the clinical characteristics of COVID-19 infection in 26 children. medRxiv [Preprint] (2020). Available at: https://www.medrxiv.org/content/10.1101/2020.03.08.20029710v1 (Accessed March 10, 2020).

29. Peng D, Zhang J, Xu Y, Liu Z, Wu P. Clinical analysis and early differential diagnosis of suspected pediatric patients with 2019 novel coronavirus infection. medRxiv [Preprint] (2020). Available at: https://europepmc.org/article/ppr/ppr149835 (Accessed April 10, 2020).

30. Wu Q, Xing Y, Shi L, Li W, Gao Y, Pan S, et al. Epidemiological and Clinical Characteristics of Children with Coronavirus Disease 2019. medRxiv [Preprint] (2020). Available at: https://www.medrxiv.org/content/10.1101/2020.03.19.20027078v2 (Accessed March 26, 2020)

31. Liu, Shijian and Yuan, Chunhui and Lin,et al. Association of Vaccinations and Clinical Manifestations in Children with COVID–19 SSRN [Preprint] (2020). Available at https://ssrn.com/abstract=3572882 or http://dx.doi.org/10.2139/ssrn.3572882 (Accessed April 23, 2020)

32. Yu H, Cai Q, Dai X, Liu X, Sun H. The clinical and epidemiological features and hints of 82 confirmed COVID-19 pediatric cases aged 0-16 in Wuhan, China. medRxiv [Preprint] (2020). Available at <https://www.medrxiv.org/content/10.1101/2020.03.15.20036319v1> (Accessed March 18, 2020)

33. Zhang C, Gu J, Chen Q, Deng N, Li J, Huang L, et al. Clinical characteristics of 34 children with coronavirus disease-2019 in the west of China: A multiple-center case series. medRxiv [Preprint] (2020). Available at https://www.medrxiv.org/content/10.1101/2020.03.12.20034686v1 (Accessed March 16, 2020)

34. Tan YP, Tan BY, Pan J, Wu J, Zeng SZ, Wei HY. Epidemiologic and clinical characteristics of 10 children with coronavirus disease 2019 in Changsha, China. J Clin Virol (2020) 127:104353. doi: 10.1016/j.jcv.2020.104353

35. Xu H, Liu E, Xie J, Smyth R, Zhou Q, Zhao R, et al. A follow-up study of children infected with SARS-CoV-2 from Western China. Ann Transl Med (2020) 8(10):623 doi: 10.21037/atm-20-3192

36. Shekerdemian LS, Mahmood NR, Wolfe KK, Riggs BJ, Ross CE, McKiernan CA, et al. Characteristics and Outcomes of Children With Coronavirus Disease 2019 (COVID-19) Infection Admitted to US and Canadian Pediatric Intensive Care Units. JAMA Pediatr [Preprint] (2020). Available at https://doi.org.10.1001/jamapediatrics.2020.1948 (Accessed May 12, 2020).

37. Liu J, Luo WJ, Deng ZH, Wang XJ, Nie L, Wang WJ, et al. Clinical and epidemiological characteristics of 91 children conformed with COVID-19. Chin J Nosocomiol (2020):1-5. doi:10.11816/cn.ni.2020200550

38. Ji TY, Chen QJ, Chen FC, Huang LJ, Chen SJ, Lv MZ, et al. Clinical characteristics and drug therapy of 4 Cases of children with corona virus disease 2019. J Pediatric Pharmacy (2020) 26(04):24-7. doi:10.13407/j.cnki.jpp.1672-108X.2020.04.008

39. Wang D, Ju XL, Xie F, Lu Y, Li FY, Huang HH, et al. Clinical analysis of 31 cases of 2019 novel coronavirus infection in children from six provinces (autonomous region) of northern China. Chin J Pediatr (2020) 58(4):E011. doi: 10.3760/cma.j.cn112140-20200225-00138

40. Zhou Y, Yang GD, Feng K, Huang H, Yun YX, Mou XY, et al. Clinical features and chest CT findings of coronavirus disease 2019 in infants and young children. Chin J Contemp Pediatr (2020) 22(3):215-20. doi:10.7499/j.issn.1008-8830.2020.03.007

41. Ma YL, Xia SY, Wang M, Zhang SM, Du WH, Chen Q. Clinical features of children with SARS-CoV-2 infection: an analysis of 115 cases. Chin J Contemp Pediatr (2020):1-4. doi:10.7499/j.issn.1008-8830.2003016

42. Tan X, Huang J, Zhao F, Zhou Y, Li JQ, Wang XY. Clinical features of children with SARS-CoV-2 infection: an analysis of 13 cases from Changsha, China. Chin J Contemp Pediatr (2020):1-5. doi:10.7499/j.issn.1008-8830.2003199

43. Feng K, Yun YX, Wang XF, Yang GD, Zheng YJ, Lin CM, et al. [Analysis of CT features of 15 Children with 2019 novel coronavirus infection]. Chin J Pediatr (2020) 58(0):E007. doi: 10.3760/cma.j.issn.0578-1310.2020.0007

44. Yang L, Li Z, Xu HR, Chang CY, Liu Z, Li CB, et al. Epidemiological and clinical characteristics of 10 children with coronavirus disease (COVID-19) in Jinan. J ShanDong Uni (Health Sci) (2020):1-4. doi: 10.6040/j.issn.1671-7554.0.2020.305

45. Jiang JY, Duan L, Xiong DX, Feng Y, Liu XJ, Xu J, et al. Epidemiological and clinical characteristics of novel coronavirus infection in children Epidemiological and clinical characteristics of novel oronavirus infection in children ：Thoughts on the diagnostic criteria of suspected cases outside Hubei Province. Chin Pediatr Emer Med (2020) 27(00): E003-E. doi: 10.3760/cma.j.issn.1673-4912.2020.0003

46. Zhang XG, Ma Y, Xiao J, Zhang ZF. Clinical characteristics of novel coronavirus pneumonia in children in Jinan. J ShanDong Uni (2020). doi: 10.6040/j.issn.1671-7554.0.2020.180

47. Wu HP, Li BF, Chen X, Hu HZ, Jiang SA, Chen H, et al. Clinical features of coronavirus disease 2019 in children aged <18 years in Jiangxi, China: an analysis of 23 cases. Chin J Contemp Pediatr (2020):1-6. doi: 10.7499/j.issn.1008-8830.2003202

48. Li Q, Peng XH, Sun ZY, Shao JP. Clinical and imaging characteristics of children with coronavirus disease 2019 (COVID-19). Radiol Practice (2020) 35(03):277-80. doi: 10.13609/j.cnki.1000-0313.2020.03.007

49. Xiong JX, Zhou CY, Zeng WB. CT findings of children with COVID-19. Chongqing Med (2020):1-4.

50. Zhong Z, Xie XZ, Huang W, Zhao W, Yu QZ, Liu J. Chest CT findings and clinical features of coronavirus disease 2019 in children. J Cent South Univ (Med Sci) (2020):1-7. doi: 10.11817/j.issn.1672-7347.2020.200206

51. Ma HJ, Shao JB, Wang YJ, Cui AG, Zheng NN, Li Q, et al. High resolution CT features of novel coronavirus pneumonia in children Chin J Radiology (2020) (04):310-1-2-3. doi:10.3760/cma.j.cn112149-20200206-00100

52. Chen J, Wang XF, Zhang PF. Asymptomatic SARS-CoV-2 infection in children: a clinical analysis of 20 cases. Chin J Contemp Pediatr (2020):1-5. doi: 10.7499/j.issn.1008-8830.2003084

53. Yang XN, Deng J, Li XF, Fang CX, Gong Y, Wang YL, et al. Distribution of traditional chinese medicine syndromes in children with coronavirus disease 2019: An analysis of 11 cases. J Guangzhou Uni Tradit Chin Med (2020):1-8. doi: 10.13359/j.cnki.gzxbtcm

54. Feng XY, Tao XW, Zeng LK, Wang WQ, Li GD. Application of pulmonary ultrasound in the diagnosis of COVID-19 pneumonia in neonates. Chin J Pediatr (2020) (05): E013-E. doi:10.3760/cma.j.cn112140-20200228-00154
