# Supplementary material for: Epidemiological and Clinical Characteristics of COVID-19 in Children: A Systematic Review and Meta-Analysis
Source: Front Pediatr. 2020 Nov 2;8:591132. doi: 10.3389/fped.2020.591132 (PMC7667131; doi:10.3389/fped.2020.591132)
Supplement: Supplementary file 3 [file Table_3.DOCX]

**Supplementary Table 3 Epidemiologic data, ICU requirement, and comorbidities of the included studies on COVID-19, 2020**

| **ID** | **Study** | **N** | **Epidemiologic history (n (%))** | | | | **Epidemiological data (days, Mean ± SD）** | | | | | **n (%)** | | |
| --- | --- | --- | --- | --- | --- | --- | --- | --- | --- | --- | --- | --- | --- | --- |
|  |  |  | **Family Cluster** | **Endemic area** | **Close contact** | **Other** | **E→O** | **O→D1** | **E→D1** | **A→D2** | **N+→N−** | **R+** | **N at ICU** | **Comorbi-dities** |
| 1 | Cai et al. | 10 | 7 (70.0) | 2 (20.0) | 8 (80.0) | 1 (10.0) | 6.5 ± 2.3 | 12.0 ± 4.6 | 11.8 ± 4.2 | 21.8 ± 4.3 | 12.0 ± 4.8 | 5(83.3) | - | - |
| 2 | Hu et al. | 6 | - | 3 (50.0) | - | - | - | - | - | 10.8 ± 5.9 | 8.0 ± 6.4 | - | - | - |
| 3 | Zhu et al. | 10 | 7 (70.0) | 3 (30.0) | 7 (70.0) | - | - | - | - | - | - | - | - | - |
| 4 | CDC COVID-19 Team | 2572 | 168/184  (91.3) | - | - | 16/184  (9.0) | - | - | - | - | - | - | 15/745  (2.0) | 80/345  (23.2) |
| 5 | Turner et al. | 6 | - | - | - | - | - | - | - | - | - | - |  | 6(100) |
| 6 | Liu et al. | 6 | - | 6 (100) | - | - | - | 4.7 ± 0.8 | - | 8.3 ± 2.0 | - | - | 1(16.7) | - |
| 7 | Dong et al. | 728 | - | 229 (31.5) | - | 499 (68.5) | - | 3.0 ± 3.0 | - | - | - | - | - | - |
| 8 | Liu et al. | 5 | 5 (100) | - | - | - | - | 2.5 ± 0.7 | 6.2 ± 3.4 | - | 8.6 ± 3.7 | - | - | - |
| 9 | Tagarro et al. | 41 | 16 (39.0) | - | 16 (39.0) | 25 (61.0) | - | - |  | - | - | - | 4(9.8) | 11(26.8) |
| 10 | Su et al. | 9 | 9 (100) | 8 (88.9) | 9 (100) | - | - | - |  | - | 12.4 ± 2.7 | 6(66.7) |  | - |
| 11 | Xu et al. | 10 | 7 (70.0) | 7 (70.0) | 4 (40.0) | - | 9.7 ± 2.7 | 2.0 ± 0.9 | 11.0 ± 2.5 | 9.0 ± 1.4 | 7.5 ± 6.4 | 8(80.0) | - | - |
| 12 | Li et al. | 5 | 4 (80.0) | 1 (20.0) | 4 (80.0) | - | - | - | - | 15.2 ± 5.0 | - | - | -- | - |
| 13 | Xia et al. | 20 | 13 (65.0) | - | - | 7 (35.0) | - | - | - | 12.9 ± 3.0 | - | - |  | 7(35.0) |
| 15 | Qiu et al. | 36 | 32 (88.9) | 12 (33.3) | - | - | - | - | - | 14.0 ± 3.0 | 10.0 ± 2.0 | - | - | - |
| 16 | Zheng et al. | 25 | 16 (64.0) | 5 (20.0) | 16 (64.0) | 4 (16.0) | - | - | - | - | - | - | 2(8.0) | 2(8.0) |
| 17 | Sun et al. | 8 | 5 (62.5) | 8 (100) | 5 (62.5) | 2 (25.0) | 6.8 ± 2.4 | 7.8 ± 5.1 | 10.3 ± 4.5 | 18.5 ± 6.1 | - | - | 3(37.5) | 3(37.5) |
| 18 | Shen et al. | 9 | 9 (100) | 2 (22.2) | 6 (66.7) | - | 7.2 ± 4.2 | 6.1 ± 6.3 | 10.9 ± 6.2 | 15.3 ± 3.9 | 13.3 ± 3.9 | - | - | - |
| 20 | Li et al. | 40 | - | - | - | - | - | - | - | - | - | - | 1(2.5) | - |
| 21 | Han et al. | 7 | 7 (100) | - | 7 (100) | - | 8.8 ± 2.6 | - | - | - | - | - | - | - |
| 22 | Du et al. | 14 | 14 (100) | - | - | - | - | 6.2 ± 6.4 | - | - | - | - | - | - |
| 23 | Wei et al. | 9 | 9 (100) | 7 (77.8) | 9 (100) | 1 (11.1) | - | 1.6 ± 0.9 | - | - | - | - | - | - |
| 24 | See et al. | 4 | - | 4 (100) | - | - | - | 2.0 ± 0.8 | 5.3 ± 3.0 | - | 13.0 ± 5.4 | - | - | - |
| 25 | Lu et al. | 171 | 154 (90.1) | 156 (91.2) | 156 (91.2) | 15 (8.8) | - | - | - | - | - | - | - | - |
| 26 | Ma et al. | 6 | 6 (100) | 5 (83.3) |  | 1 (16.7) | - | - | - | - | 10.5 ± 3.8 | 6(100) | - | - |
| 28 | Tang et al. | 26 | - | 26 (100) | - | - | - | - | - | 13.6 ± 1.0 |  | - | - | - |
| 29 | Peng et al. | 35 | 29 (82.9) | - | - | - | - | - | - |  |  | - | - | - |
| 30 | Wu et al. | 74 | 65/68  (95.6) | - | -- | - | - | - | - | 11.0 ± 0.8 |  | - | - | - |
| 32 | Yu et al. | 82 | -- | 74 (90.2) | 74 (90.2) | - | - | - | - | - | - | - | 8(9.8) | 7(8.5) |
| 33 | Zhang et al. | 34 |  | 18 (52.4) | - | - | 14.5 ± 13.0 | 2.0 ± 0.6 | 16.7 ± 11.6 | - | - | - |  | 6(17.6) |
| 34 | Tan et al. | 10 | 10 (100) | 4 (40.0) | - | - | 14.6 ± 4.9 | 2.4 ± 1.3 | 15.3 ± 5.8 | 17.2 ± 4.9 | 8.1 ± 4.9 | 4(40.0) |  | - |
| 35 | Xu et al. | 32 | 29 (91.6) | 12 (37.5) | - | 2 (6.3) | - | - | - | - | 15.7 ± 7.1 | - | - | 3(9.4) |
| 36 | Shekerdemian et al. | 48 | - | - | - | - | - | - | - | 8.0 ± 6.7 | - | -- | - | 40(83.3) |
| 37 | Liu et al. | 91 | 72 (79.1) | 91 (100) | 72 (79.1) | - | - | - | - | 15.0 ± 2.2 | -- | - | - | 9(9.9) |
| 38 | Ji et al. | 4 | 3 (75.0) |  | 4 (100) | - | - | 4.8 ± 2.8 | - | - |  | - | - | -- |
| 39 | Wang et al. | 31 | 28 (90.3) | 9 (29.0) | 22 (71.0) | -- | - | - | - | - | - |  | - | - |
| 40 | Zhou et al. | 9 | 9 (100) | 3 (33.3) | 9 (100) | - | -- | -- | - | - | - | - | - | - |
| 41 | Ma et al. | 115 | 105 (91.3) | 115 (100) | 105 (91.3) | - | - |  | - | - | - | - | - | 3(2.6) |
| 42 | Tan et al. | 13 | 13 (100) | 3 (23.1) | - | - | 11.3 ± 3.7 | - | - | - | 13.1 ± 5.6 | -- |  | - |
| 43 | Feng et al. | 15 | - | 3 (20.0) | 12 (80.0) | - | - | - | - | 4.0 ± 2.9 | - | - | - | - |
| 44 | Yang et al. | 10 | 10 (100) | 4 (40.0) | 10 (100) | - | - | 2.1 ± 1.9 | - | 15.3 ± 1.5 | -- | - | - | - |
| 45 | Jiang et al. | 6 | 6 (100) | 3 (50.0) | 3 (50.0) | - | - |  | - | -- | - | - | - | - |
| 46 | Zhang et al. | 10 | 10 (100) | - | - | -- | - | 5.3 ± 5.8 | - |  | - | - | - | - |
| 47 | Wu et al. | 23 | 17 (73.9) | 7 (30.4) | - | - | - | - | - | 14.8 ± 4.3 | 13.5 ± 4.5 | - | - | - |
| 48 | Li et al. | 30 | 16 (53.3) | 30 (100) | 17 (56.7) | - | - | -- | - | - | - | - | - | - |
| 49 | Xiong et al. | 6 | 6 (100) | - | - | - | - |  | - | -- | - | - | - | - |
| 50 | Zheng et al. | 9 | 9 (100) | - | - | - | - | - | - | - | - | - | - | - |
| 51 | Ma et al. | 22 | 17 (72.3) | 22 (100) | 17 (72.3) | - | - | - | - | 10.0 ± 2.5 | - | - | - | - |
| 52 | Chen et al. | 20 | 19 (95.0) | 10 (50.0) | - | - | - | - | - | - | - | - | - | - |
| 54 | Feng et al. | 5 | 5 (100) | 5 (100) | 5 (100) | - | - | - | -- | 15.7 ± 0.6 | - | - | - | - |

E, exposure to index patient; O, symptom onset; A, admission to hospital; D1, time of diagnose; D2, discharge from hospital; N+, nucleic acid in respiratory swabs test positive; N−, nucleic acid test in respiratory swabs negative; R+, nucleic acid test in stool test positive. E→O, time between exposure and symptom onset (days); O→D1, time between symptom onset and diagnosis; E→D1, time between exposure and diagnosis; A→D2, duration of hospitalization; N+→N−, duration of virus shedding in respiratory swabs.
