# Supplementary material for: Epidemiological and Clinical Characteristics of COVID-19 in Children: A Systematic Review and Meta-Analysis
Source: Front Pediatr. 2020 Nov 2;8:591132. doi: 10.3389/fped.2020.591132 (PMC7667131; doi:10.3389/fped.2020.591132)
Supplement: Supplementary file 4 [file Table_4.DOCX]

**Supplementary Table 4 Clinical characteristics of the included studies on COVID-19, 2020**

| **ID** | **Study** | **N** | **Severity of illness (n (%))** | | | | | **n (%)** | | | | | | | | |
| --- | --- | --- | --- | --- | --- | --- | --- | --- | --- | --- | --- | --- | --- | --- | --- | --- |
|  |  |  | **Asymptomatic** | **Mild** | **Moderate** | **Severe** | **Critical.** | **Fever** | **<38.01°C** | **38.01-39°C** | **>39.0°C** | **Cough** | **Fever and cough** | **Shortness of breath** | **Headache**  **/dizziness** | **Nasal congestion** |
| 1 | Cai et al. | 10 | - | - | - | - | - | 8 (80) | 2 (20) | 5 (50) | 1 (10) | 6 (60) | - | - | - | 3 (30) |
| 2 | Hu et al. | 6 | 5 (83.3) | 1 (16.7) | - | - | - | 1 (16.7) | - | - | - | - | - | - | - | - |
| 3 | Zhu et al. | 10 | 3 (30) | 7 (70) | - | - | - | 4 (40) | - | - | - | 3 (30) | - | - | 2 (20) | - |
| 4 | CDC COVID-19 Team | 291 | 1 (0.3) | 251 (86.3) | 39 (13.4) | - | - | 163 (56) | - | - | - | 158 (54.3) | - | 39 (13.4) | 81 (27.8) | - |
| 5 | Turner et al. | 6 | - | 6 (100) | - | - | - | 3 (50) | - | - | - | 4 (66.7) | - | - | - | 1 (16.7) |
| 6 | Liu et al. | 6 | - | 2 (33.3) | 3 (50) | 1 (16.7) | - | 6 (100) | - | - | - | 6 (95.8) | - | - | - | - |
| 7 | Dong et al. | 727 | 94 (12.9) | 314 (43.2) | 298 (41) | 18 (2.5) | 3 (0.4) |  | - | - | - |  | - | - | - | - |
| 8 | Liu et al. | 5 | 3 (60) | 2 (40) | - | - | - | 2 (40) | 2 (40) | - | - | 2 (40) | - | - | - | - |
| 9 | Tagarro et al. | 41 | - | - | - | - | - | 11 (26.8) | - | - | - |  | - | - | - | - |
| 10 | Su et al. | 9 | 6 (66.7) | 3 (33.3) | - | - | - | 2 (22.2) | 1 (11.1) | - | 1 (11.1) | 1 (11.1) | - | - | - | - |
| 11 | Xu et al. | 10 | 1 (10) | 9 (90) | - | - | - | 7 (70) | 2 (20) | 3 (30) | 2 (20) | 5 (50) | - | - | - | 2 (20) |
| 12 | Li et al. | 5 | 4 (80) | 1 (20) | - | - | - | 1 (20) | - | - | - | 1 (20) | - | - | - | - |
| 13 | Xia et al. | 20 | 2 (10) | 18 (90) | - | - | - | 12 (60) | - | - | - | 13 (65) | - | - | - | - |
| 14 | Liu et al. | 4 | - | - | - | - | - | 3 (75) | - | - | - | 3 (75) | - | - | - | - |
| 15 | Qiu et al. | 36 | 10 (27.8) | 7 (19.4) | 19 (52.8) | - | - | 13 (36.1) | - | - | - | 7 (19.4) | - | - | 3 (8.3) | - |
| 16 | Zheng et al. | 25 | - | 8 (32) | 15 (60) | - | 2 (8) | 13 (52) | - | - | - | 11 (44) | - | - | - | 2 (8) |
| 17 | Sun et al. | 8 | - | - | - | 5 (62.5) | 3 (37.5) | 6 (75) | - | - | - | 6 (75) | - | - | 1 (12.5) | - |
| 18 | Shen et al. | 9 | 2 (22.2) | 7 (77.8) | - | - | - | 4 (44.4) | 3 (33.3) | - | 1 (11.1) | 1 (11.1) | - | - | - | - |
| 19 | Li et al. | 22 | - | - | - | - | - | 14 (63.6) | - | - | - | 13 (59.1) | - | - | - | - |
| 20 | Li et al. | 40 | - | - | - | - | - | 21 (52.5) | - | - | - | 27 (67.5) | - | - | - | - |
| 21 | Han et al. | 7 | - | 7 (100) | - | - | - | 5 (71.4) | - | - | - | 5 (71.4) | - | 3 (42.9) | - | - |
| 22 | Du et al. | 14 | - | 3 (21.4) | 11 (78.6) | - | - | 5 (35.7) | - | - | - | 3 (21.4) | - | - | 1 (7.1) | - |
| 23 | Wei et al. | 9 | - | - | - | - | - | 4/7 (57.1) | - | - | - | 2/7 (28.6) | - | - | - | - |
| 24 | See et al. | 4 | 1 (25) | 3 (75) | - | - | - | 2 (50) | - | - | - | 1 (25) | - | - | - | - |
| 25 | Lu et al. | 171 | 27 (15.8) | 12 (7) | 129 (75.4) | - | 3 (1.8) | 71 (41.5) | 16 (9.4) | 39 (22.8) | 16 (9.4) | 83 (48.5) | - | - | 9 (5.3) | - |
| 26 | Ma et al. | 6 | 4 (66.7) | 2 (33.3) | - | - | - | 2 (33.3) | 1 (16.7) | 1 (16.7) | - | - | - | - | - | - |
| 27 | Wang et al. | 2141 | 94 (4.4) | 1091 (51) | 831 (38.8) | 112 (5.2) | 13 (0.6) | - | - | - | - | - | - | - | - | - |
| 28 | Tang et al. | 26 | - | 8 (30.8) | 18 (69.2) | - | - | 11 (42.3) | - | - | - | 12 (46.2) | - | - | - | - |
| 29 | Peng et al. | 35 | 16 (45.7) | - | - | - | - | 12 (34.3) | - | - | - |  | - | - | - | - |
| 30 | Wu et al. | 74 | 20 (27) | 24 (32.4) | 29 (39.2) | 1 (1.4) | - | 20 (27) | 2 (2.7) | 10 (13.5) | 8 (10.8) | 24 (32.4) | - | - | 2 (2.7) | - |
| 31 | Liu et al. | 248 | 92 (37.1) | 147 (59.3) | 9 (3.6) | - | - | 93 (37.5) | - | - | - | 84 (33.9) | - | 4 (1.6) | 1 (0.4) | - |
| 32 | Yu et al. | 82 | 8 (9.8) | 63 (76.8) | - | 3 (3.7) | 8 (9.8) | 14 (17.1) | - | - | - | 14 (17.1) | 37 (45.1) | 2 (2.4) | - | - |
| 33 | Zhang et al. | 34 | - | - | - | - | - | 26 (76.5) | - | - | - | 20 (58.8) | - | - | - | - |
| 34 | Tan et al. | 10 | 2 (20) | 3 (30) | 5 (50) | - | - | 4 (40) | 3 (30) | - | 1 (10) | 3 (30) | - | - | - | - |
| 35 | Xu et al. | 32 | 11 (34.4) | 21 (65.6) | - | - | - | 8 (38.1) | - | - | - | 12 (57.1) | 6 (28.6) | - | 2 (9.5) | 3 (14.3) |
| 36 | Shekerdemian et al. | 48 | 1 (2.1) | 13 (27.1) | 1 (2.1) | 16 (33.3) | 17 (35.4) | - | - | - | - | - | - | - | - | - |
| 37 | Liu et al. | 91 | 7 (7.7) | 46 (50.5) | 31 (34.1) | 6 (6.6) | 1 (1.1) | 56 (61.5) | 17 (18.7) | 29 (31.9) | 10 (11) | 51 (56) | - | - | 2 (2.2) | - |
| 38 | Ji et al. | 4 | - | 1 (25) | 3 (75) | - | - | 2 (50) | 1 (25) | - | - | 1 (25) | - | - | - | 1 (25) |
| 39 | Wang et al. | 31 | 4 (12.9) | 13 (41.9) | 14 (45.2) | - | - | 20 (64.5) | 10 (32.3) | 9 (29) | 1 (3.2) | 14 (45.2) | - | - | 3 (9.7) | - |
| 40 | Zhou et al. | 9 | 5 (55.6) | 4 (44.4) | - | - | - | 4 (44.4) | - | - | - | 2 (22.2) | - | - | - | - |
| 41 | Ma et al. | 115 | 61 (53) | 51 (44.3) | - | - | 3 (2.6) | 29 (25.2) | - | - | - | - | - | - | - | - |
| 42 | Tan et al. | 13 | 2 (15.4) | 5 (38.5) | 5 (38.5) | 1 (7.7) | - | 6 (46.2) | - | - | - | 6 (46.2) | - | - | - | - |
| 43 | Feng et al. | 15 | - | 3 (20) | 12 (80) | - | - | 5 (33.3) | - | - | - | 1 (6.7) | - | - | - | 1 (6.7) |
| 44 | Yang et al. | 10 | - | 3 (30) | 7 (70) | - | - | 4 (40) | - | - | - | 1 (10) | - | - | - | - |
| 45 | Jiang et al. | 6 | 2 (33.3) | 2 (33.3) | 2 (33.3) | - | - | 3 (50) | - | - | - | 2 (33.3) | - | - | - | - |
| 46 | Zhang et al. | 10 | - | 3 (30) | 7 (70) | - | - | 4 (40) | - | - | - | 1 (10) | 1 (10) | - | - | - |
| 47 | Wu et al. | 23 | 3 (13) | 6 (26.1) | 14 (60.9) | - | - | 16 (69.6) | 11 (47.8) | 2 (8.7) | 3 (13) | 11 (47.8) | 8 (34.8) | - | 1 (4.3) | - |
| 48 | Li et al. | 30 | - | - | 29 (96.7) | 1 (3.3) | - | 25 (83.3) | - | - | - | 20 (66.7) | - | - | - | - |
| 49 | Xiong et al. | 6 | 2 (33.3) | 2 (33.3) | 2 (3.3) | - | - | 3 (50) | - | - | - | 4 (66.7) | - | - | - | - |
| 50 | Zheng et al. | 9 | - | 3 (33.3) | 6 (66.7) | - | - | 2 (22.2) | 1 (11.1) | - | 1 (11.1) | 5 (55.6) | 1 (11.1) | - | - | 1 (11.1) |
| 51 | Ma et al. | 22 | 2 (9.1) | 1 (4.5) | 18 (81.8) | - | 1 (4.5) | 13 (59.1) | 8 (36.4) | 3 (13.6) | 2 (9.1) | 7 (31.8) | - | - | - | - |
| 52 | Chen et al. | 20 | - | 4 (20) | 16 (80) | - | - | - | - | - | - | - | - | - | - | - |
| 53 | Yang et al. | 11 | - | 3 (27.3) | 8 (72.7) | - | - | 7 (63.6) | - | - | - | 5 (45.5) | - | - | - | 3 (27.3) |
| 54 | Feng et al. | 5 | - | 2 (40) | 3 (60) | - | - | 2 (40) | - | - | - | - | - | - | - | - |

**Supplementary Table 3 Clinical characteristics of the included studies on COVID-19, 2020 (continued).**

| **ID** | **Study** | **N** | **n (%)** | | | | | | | | | | | | |
| --- | --- | --- | --- | --- | --- | --- | --- | --- | --- | --- | --- | --- | --- | --- | --- |
|  |  |  | **Sneezing** | **Rhinorrhea** | **Sore throat** | **Sputum** | **Dyspnea** | **Upper airway infections** | **Wheezing** | **Diarrhea** | **Constipation** | **Nausea** | **Fatigue** | **Anore-xia** | **Abdominalpain** |
| 1 | Cai et al. | 10 | 2 (20) | 2 (20) | 4 (40) | - | - | - | - | - | - | - | - | - | - |
| 2 | Hu et al. | 6 | - | - | - | - | - | - | - | - | - | - | - | - | - |
| 3 | Zhu et al. | 10 | - | - | - | - | - | - | - | - | - | - | - | - | - |
| 4 | CDC COVID-19 Team | 291 | - | 21 (7.2) | 71 (24.4) | - | - | - | - | 37 (12.7) | - | 31 (10.7) | - | - | 17 (5.8) |
| 5 | Turner et al. | 6 | - | - | - | - | - | - | - | - | - | - | 2 (33.3) | - | - |
| 6 | Liu et al. | 6 | - | 1 (16.7) | - | - | 1 (16.7) | - | 2 (33.3) | - | - | 4 (66.7) | - | - | - |
| 7 | Dong et al. | 727 | - | - | - | - | - | - | - | - | - | - | - | - | - |
| 8 | Liu et al. | 5 | - | - | - | - | - | - | 1 (2.4) |  | - | - | - | - | - |
| 9 | Tagarro et al. | 41 | - | - | - | - | - | 14 (34.1) | - | -- | - | 2 (4.9) | - | - | - |
| 10 | Su et al. | 9 | - | - | - | - | - | - | - | - | - | - | - | - | - |
| 11 | Xu et al. | 10 | - | 2 (20) | 4 (40) | - | - | - | - | 3 (30) | - | - | - | - | - |
| 12 | Li et al. | 5 | - | 1 (20) | 1 (20) | 1 (20) | - | - | - | - | - | - | - | - | - |
| 13 | Xia et al. | 20 | - | 3 (15) | 1 (5) | - | 2 (10) | - | - | 3 (15) | - | 2 (10) | 1 (5) | - | - |
| 14 | Liu et al. | 4 | - | - | - | - | - | - | - | - | - | - | 1 (25) | - | - |
| 15 | Qiu et al. | 36 | - | - | 2 (5.6) | - | 1 (2.8) | - | - | - | - | 2 (5.6) | - | - | - |
| 16 | Zheng et al. | 25 | - | - | - | - | 2 (8) | 8 (32) | - | 3 (12) | - | 2 (8) | - | - | 2 (8) |
| 17 | Sun et al. | 8 | - | - | - | 4 (50) | 8 (100) | - | - | 3 (37.5) | 1 (12.5) | 4 (50) | 1 (12.5) | - | - |
| 18 | Shen et al. | 9 | - | - | 1 (11.1) | - | - | - | - | 2 (22.2) | - | - | - | - | - |
| 19 | Li et al. | 22 | - | - | - | - | - | - | - | - | - | - | - | - | - |
| 20 | Li et al. | 40 | - | 2 (5) | 2 (5) | - | 1 (2.5) | - | - | 2 (5) | - | - | 4 (10) | - | - |
| 21 | Han et al. | 7 | - | - | 1 (14.3) | - | - | - | - | - | - | 4 (57.1) | - | - | - |
| 22 | Du et al. | 14 | - | - | 1 (7.1) | 1 (7.1) | 0 (0) | - | - | - | - | 0 (0) | 1 (7.1) | - | - |
| 23 | Wei et al. | 9 | - | 1/7 (14.3) | - | 1/7 (14.3) | - | - | - |  | - | - | - | - | - |
| 24 | See et al. | 4 | 1 (25) | - | - | - | - | 1 (25) | - | -- | - | - | - | - | - |
| 25 | Lu et al. | 171 | 13 (7.6) | - | 79 (46.2) | - | - | 15 (8.8) | 11 (6.4) | - | 13 (7.6) | - | - | - | - |
| 26 | Ma et al. | 6 | - | - | - | - | - | - | - | - | - | - | - | - | - |
| 27 | Wang et al. | 2143 | - | - | - | - | - | - | - | - | - | - | - | - | - |
| 28 | Tang et al. | 26 | - | 2 (7.7) | - | - | - | - | - | 2 (7.7) | - | 2 (7.7) | - | - | - |
| 29 | Peng et al. | 35 | - | - | - | - | 13 (37.1) | - | - | - | - | - | - | - | - |
| 30 | Wu et al. | 74 | - | - | 0 (0) | 2 (2.7) | 2 (2.7) | 24 (32.4) | - | 3 (4.1) | - | - | 5 (6.8) | 3 (4.1) | - |
| 31 | Liu et al. | 248 | - | - | 3 (1.2) | 79 (31.9) | - | - | - | 6 (2.4) | - | 7 (2.8) | - | 4 (1.6) | 3 (1.2) |
| 32 | Yu et al. | 82 | - | - | - | - | - | - | - | - | - | 7 (8.5) | - | - | - |
| 33 | Zhang et al. | 34 | - | - | - | 7 (20.6) | 3 (8.8) | - | - | 4 (11.8) | - | 4 (11.8) | - | - | - |
| 34 | Tan et al. | 10 | - | - | - | - | - | - | - | - | 1 (10) | 1 (10) | - | - | 1 (10) |
| 35 | Xu et al. | 32 | - | 5/21 (23.8) | 3/21 (14.3) | - | - | - | - | - | - | - | 2/21 (9.5) | - | - |
| 36 | Shekerdemian et al. | 48 | - | - | - | - | - | - | - | - | - | - | - | - | - |
| 37 | Liu et al. | 91 | - | - | - | - | - | - | - | 5 (5.5) | - | 5 (5.5) | 14 (15.4) | - | - |
| 38 | Ji et al. | 4 | - | 1 (25) | - | - | 1 (25) | - | - | - | - | - | - | - | - |
| 39 | Wang et al. | 31 | - | 2 (6.5) | 2 (6.5) | 6 (19.4) | - | - | - | 3 (9.7) | - | 2 (6.5) | 3 (9.7) | - | - |
| 40 | Zhou et al. | 9 | - | 1 (11.1) | - | - | - | - | - | - | - | - | - | - | - |
| 41 | Ma et al. | 115 | - | - | - | - | - | 47 (40.9) | - | 3 (2.6) | - | - | - | - | - |
| 42 | Tan et al. | 13 | - | - | 2 (15.4) | - | - | - | - | 2 (15.4) | - | 1 (7.7) | - | - | 1 (7.7) |
| 43 | Feng et al. | 15 | - | - | - | - | - | - | - | - | - | - | - | - | - |
| 44 | Yang et al. | 10 | - | - | - | - | - | - | - | - | - | - | - | - | - |
| 45 | Jiang et al. | 6 | - | - | 1 (16.7) | - | - | - | - | 1 (16.7) | - | - | - | - | - |
| 46 | Zhang et al. | 10 | - | 1 (10) | - | - | - | - | - | - | - | - | - | - | - |
| 47 | Wu et al. | 23 | - | 1 (4.3) | 1 (4.3) | 4 (17.4) | - | - | 4 (17.4) | 2 (8.7) | - | - | - | - | 1 (4.3) |
| 48 | Li et al. | 30 | - | 2 (6.7) | 1 (3.3) | - | - | - | - | 2 (6.7) | - | 2 (6.7) | 2 (6.7) | - | - |
| 49 | Xiong et al. | 6 | - | - | 1 (16.7) | - | - | - | - | 1 (16.7) | - | - | - | 1 (16.7) | - |
| 50 | Zheng et al. | 9 | - | - | 1 (11.1) | - | - | - | - | - | - | - | - | - | - |
| 51 | Ma et al. | 22 |  | 3 (13.6) | 1 (4.5) | 2 (9.1) | 1 (4.5) | - | - | 1 (4.5) | - | - | 1 (4.5) | 1 (4.5) | - |
| 52 | Chen et al. | 20 | - | - | - | -- |  | - | - | - | - | - | - | - | - |
| 53 | Yang et al. | 11 | - | 3 (27.3) | 2 (18.2) | 3 (27.3) | - | - | - | - | - | - | - | - | - |
| 54 | Feng et al. | 5 | 1 (20.0) |  |  | - | - | - | - | 1 (20) | - | - | 3 (60) | 3 (60) | - |
