# Supplementary material for: Epidemiological and Clinical Characteristics of COVID-19 in Children: A Systematic Review and Meta-Analysis
Source: Front Pediatr. 2020 Nov 2;8:591132. doi: 10.3389/fped.2020.591132 (PMC7667131; doi:10.3389/fped.2020.591132)
Supplement: Supplementary file 5 [file Table_5.DOCX]

**Supplementary Table 5 Laboratory characteristics of the included studies on COVID-19, 2020**

| **ID** | **Study** | **N** | **n (%)** | | | | | | | | | | | |
| --- | --- | --- | --- | --- | --- | --- | --- | --- | --- | --- | --- | --- | --- | --- |
|  |  |  | **Leukopenia** | **Leukocytosis** | **Lymphopenia** | **Lymphocytosis** | **Neutrophilia** | **Neutropenia** | **Decreased Hb** | **Decreased Albumin** | **High PLT** | **High CRP** | **High CK-MB** | **High D-dimer** |
| 1 | Cai et al. | 10 | 1 (10) | 3 (30) | **-** | 1 (10) | 1 (10) | 3 (30) | **-** | **-** | 2 (20.0) | 3 (30.0) | 5 (50.0) | 2 (20.0) |
| 2 | Hu et al. | 6 | **-** | **-** | 1 (16.7) | **-** | **-** | **-** | **-** | **-** | **-** | **-** | **-** | **-** |
| 3 | Zhu et al. | 10 | **-** | **-** | **-** | **-** | **-** | **--** | **-** | **-** | **-** | **-** | **-** | **-** |
| 6 | Liu et al. | 6 | 4 (66.7) | **-** | 6 (100) | **-** | **-** | 3 (50.0) | 1 (16.7) | **-** | **-** | 5 (83.3) | **-** | 3 (50.0) |
| 9 | Tagarro et al. | 41 | **-** | **-** | **-** | **-** | **-** | **-** | **-** | **-** | **--** | **-** | **-** | **-** |
| 10 | Su et al. | 9 | 2 | 1 (11.1) | **-** | **-** | **-** | **-** | **-** | **-** | **-** | **-** | 6 (66.7) | **-** |
| 11 | Xu et al. | 9 | 3 (33.3) | **-** | 3 (33.3) | 2 (22.2) | 1 (11.1) | 4 (44.4) | **-** | 2 (22.2) | **-** | 3 (33.3) | **-** | **-** |
| 12 | Li et al. | 5 | **-** | 2 (40.0) | **-** | **-** | **-** | **-** | **-** | **-** | **-** | 1 (20.0) | **-** | **-** |
| 13 | Xia et al. | 20 | 4 (20.0) | 2 (10.0) | 7 (35.0) | 3 (15.0) | **-** |  |  |  | **-** | 9 (45.0) | 15 (75.0) | **-** |
| 14 | Liu et al. | 4 | 1 (25.0) | **-** | 1 (25.0) | 2 (50.0) | **-** | 2 (50.0) | **-** | **-** | **-** | 1 (25.0) | **-** | **-** |
| 15 | Qiu et al. | 36 | 7 (19.4) | **-** | 11 (30.6) | **-** | **-** | **-** | **-** | **-** | **-** | 1 (2.8) | 11 (30.6) | 3 (8.3) |
| 16 | Zheng et al. | 25 | **-** | **-** | 10 (40.0) | **-** | **-** | **-** | 1 (4.0) | 2 (8.0) | **-** | 1 (4.0) | **-** | **-** |
| 17 | Sun et al. | 8 | 1 (12.5) | 2 (25.0) | 1 (12.5) | 1 (12.5) | 2 (25.0) | 1 (12.5) | 3 (37.5) | **-** | 2 (25.0) | 5 (62.5) | **-** | 2 (25.0) |
| 18 | Shen et al. | 9 | **-** | 1 (11.1) | **-** | 1 (11.1) | **-** | **-** | **-** | **-** | **-** | 1 (11.1) | **-** | **-** |
| 20 | Li et al. | 40 | **-** | **-** | 1 (2.5) | **-** | **-** | **--** | **-** | **-** | **-** |  | **-** | **-** |
| 21 | Han et al. | 7 | **-** | 2 (28.6) | **-** | 1 (14.3) | **-** | **-** | **-** | **-** | 1 (14.3) | 2 (28.6) | **-** | 2 (28.6) |
| 22 | Du et al. | 14 | 4 (28.6) | **-** | 1 (7.1) | **-** | **-** | 9 (64.0) | **-** | **-** | 2 (14.3) | 1 (7.1) | **-** | 5 (35.7) |
| 28 | Tang et al. | 26 | 13 (50.0) | 4 (15.4) | 1 (3.8) | 25 (96.2) | **-** | **-** | **-** | **-** | **-** | 5 (19.2) | **-** | **-** |
| 29 | Peng et al. | 35 | **--** | **-** |  | **--** | **-** |  | **-** | **-** | **-** | **-** | **-** | **-** |
| 30 | Wu et al. | 74 | 4 (5.4) | 19 (25.7) | 4 (5.4) | 6 (8.1) | **-** | **--** | **-** | **-** | **-** | 13 (17.6) | **-** | **-** |
| 31 | Liu et al. | 248 | **-** | **-** | **--** | **-** | **-** | **-** | **-** | **-** | **-** | 42 (16.9) | **-** | **-** |
| 32 | Yu et al. | 82 | 4 (4.9) | 21 (25.6) | 48 (58.5) | 16 (19.5) | 10 (12.2) | 36 (43.9) | 19 (23.2) | 30 (36.6) | 11 (13.4) | 23 (28.0) | 37 (45.1) | **-** |
| 33 | Zhang et al. | 34 | **-** | **-** | **-** | 17 (50.0) | **-** | **-** | **-** | **-** | **-** | 17 (50.0) | **-** | **-** |
| 34 | Tan et al. | 10 | **-** | 1 (10.0) | **-** | 1 (10.0) | **-** | **-** | **-** | **-** | **-** | 1 (10.0) | 1 (10.0) |  |
| 35 | Xu et al. | 32 | 1 (3.1) | 2 (6.3) | 2 (6.3) | 9 (28.1) | **-** | 8 (0.25) | **-** | 1 (3.1) | **-** | 3 (12.0) | **-** | 6 (24.0) |
| 37 | Liu et al. | 91 | **-** | **-** | **-** | **-** | **-** | **-** | **-** | **-** | **-** | 32 (35.2) | **-** | **-** |
| 38 | Ji et al. | 4 | **-** | **-** | **-** | 2 (50.0) | **-** | 2 (50.0) | **-** | **-** | **-** |  | 2 (50.0) | **-** |
| 39 | Wang et al. | 31 | 2 (6.5) | 3 (9.7) | 2 (6.5) | 4 (12.9) | **-** | **-** | **-** | **-** | 2 (6.5) | 3 (10.0) | 4 (14,8) | 2 (9.5) |
| 40 | Zhou et al. | 9 | **-** | 2 (22.2) |  | 6 (66.7) | **--** | **-** | **-** | **-** | **-** | 3 (42.9) | **-** | **-** |
| 41 | Ma et al. | 115 | 23 (20.0) | 4 (3.5) | 15 (13.0) | 40 (34.8) | 32 (27.8) | 6 (5.2) | **-** | **-** | **-** | **-** | 34 (29.6) | **-** |
| 42 | Tan et al. | 13 | **-** | 2 (15.4) | **-** | 1 (7.7) | **-** | 3 (23.1) | **-** | **-** | **-** | **-** | **-** | **--** |
| 43 | Feng et al. | 15 | 8 (53.3) | **-** | **-** | **-** | **-** | **--** | **-** | **-** | **--** | **-** | **-** | **-** |
| 46 | Zhang et al. | 10 | 6 (60.0) | **-** | 1 (10.0) | 5 (20.0) | **-** | **-** | **-** | **-** | **-** | **-** | 8 (80.0) | **-** |
| 47 | Wu et al. | 23 | 3 (13.0) | 2 (8.7) | 2 (8.7) | **-** | **-** | **-** | **-** | **-** | 4 (17.4) | **-** | **-** | 2 (10.5) |
| 48 | Li et al. | 30 | 11 (36.7) | **-** | 10 (33.3) | **-** | **-** | **-** | **-** | **-** | **-** | **-** | **--** | **-** |
| 49 | Xiong et al. | 6 | **-** | **-** | **-** | **-** | **-** | **-** | **-** | **-** | **-** | **-** | **-** | **-** |
| 50 | Zheng et al. | 9 | 1 (11.1) | 1 (11.1) | 1 (11.1) | 2 (22.2) | 1 (11.1) | 5 (55.6) | **-** | **-** | **-** | **-** | **-** | 2 (25.0) |
| 51 | Ma et al. | 22 | 4 (18.2) | **-** | 2 (9.1) | 2 (9.1) | **-** | **-** | **-** | **-** | **-** | 9 (40.9) | **-** | **-** |
| 52 | Chen et al. | 20 | 2 (10.0) | 5 (25.0) | 1 (5.0) | **-** | **-** | **-** | **-** | **-** | **-** | **-** | **-** | **-** |

**Supplementary Table 4 Laboratory characteristics of the included studies on COVID-19, 2020 (continued).**

| **ID** | **Study** | **N** | **n (%)** | | | | | | | | | | | | | | | | |
| --- | --- | --- | --- | --- | --- | --- | --- | --- | --- | --- | --- | --- | --- | --- | --- | --- | --- | --- | --- |
|  |  |  | **High PCT** | **High ALT** | **High AST** | **High LDH** | **High CK** | **High Creatin-ine** | **High Bilirubin** | **High ESR** | **High PT** | **High IL-6** | **High IL-10** | **Flu A** | **Flu B** | **MP** | **RSV** | **EB** | **ADV** |
| 1 | Cai et al. | 10 | **-** | 1 (10.0) | 2 (20.0) | 3 (30.0) | **-** | **-** | **-** | **-** | **-** | **-** | **-** | **-** | **-** | **-** | **-** | **-** | **-** |
| 2 | Hu et al. | 6 | 2 (33.3) | 2 (33.3) | **-** | 2 (33.3) | **-** | **-** | **-** | **-** | **-** | **-** | **-** | **-** | **-** | **-** | **-** | **-** | **-** |
| 3 | Zhu et al. | 10 | **-** | 3 (30.0) | **-** | **-** | **--** | **-** | **-** | **-** | **-** | **-** | **-** | **-** | **-** | **-** | **-** | **-** | **-** |
| 6 | Liu et al. | 6 | **-** | 1 (16.7) | 4 (66.7) | 2 (33.3) | **-** | **-** | **-** | 2 (33.3) | **-** | **-** | **-** |  | **--** | **-** | **-** | **-** | **-** |
| 9 | Tagarro et al. | 41 | **-** | **-** | **-** | **-** | **-** | **-** | **-** | **-** | **-** | **-** | **-** | **-** | 2 (4.9) | **-** | **-** | **-** | **-** |
| 10 | Su et al. | 9 | **-** | **-** | **-** | **-** | **-** | **-** | **-** | **-** | **-** | **-** | **-** | **-** | **-** | **-** | **-** | **-** | **-** |
| 11 | Xu et al. | 9 | 5 (55.6) | 1 (11.1) | 2 (22.2) | 2 (22.2) | **-** | **-** | **-** | 3 (42.9) | **-** | 5 (55.6) | **-** | **-** | **-** | **-** | **-** | **-** | **-** |
| 12 | Li et al. | 5 | **-** | **-** | **-** | **-** | **-** | **-** | **-** | **-** | **-** | **-** | **-** | **-** | **-** | **-** | **-** | **-** | **-** |
| 13 | Xia et al. | 20 | 16 (80.0) | 5 (25.0) | **-** | **-** | **-** | **-** | **-** | **-** | **-** | **-** | **-** | 1 (5.0) | 2 (10.0) | 4 (20.0) | 1 (5.0) | **-** | **-** |
| 14 | Liu et al. | 4 | **-** | **-** | **-** | **-** | **-** | **-** | **-** | **-** | **-** | **-** | **-** | **-** | **-** | **-** | 1 (25.0) | **-** | **-** |
| 15 | Qiu et al. | 36 | 6 (16.7) | 2 (55.6) | 3 (8.3) |  | 1 (2.8) |  | **-** | **-** | **-** | **-** | **-** | **-** | **-** | **-** | **-** | **-** | **-** |
| 16 | Zheng et al. | 25 | **-** | **-** | **-** | 2 (8.0) | 2 (8.0) | 1 (4.0) | **-** | **-** | 2 (8.0) | **-** | **-** | **-** | 2 (8.0) | 3 (12.0) | **-** | **-** | **-** |
| 17 | Sun et al. | 8 | 5 (62.5) | 4 (50.0) |  | 5 (62.5) | 2 (25.0) | 2 (25.0) | **-** | **-** | **-** | 2 (28.6) | 5 (71.4) | 1 (12.5) | **-** | **-** | **-** | **-** | **-** |
| 18 | Shen et al. | 9 | **-** | **-** | 2 (22.2) | 1 (11.1) | **-** | **-** | **-** | 4 (44.4) | **-** | **-** | **-** | **-** | **-** | **-** | **-** | **-** | **-** |
| 20 | Li et al. | 40 | **-** | **-** | **-** | **-** | **-** | **-** | **-** | **-** | **-** | 6 (15.0) | 10 (25.0) | **-** |  | 13 (32.5) | **-** | **-** | 1 (2.5) |
| 21 | Han et al. | 7 | 3 (43.9) | 1 (14.3) | 3 (42.9) | 2 (28.6) | 4 (57.1) | **-** | 1 (14.3) | 3 (42.9) | 3 (42.9) | **-** | **-** | **-** | **-** | **-** | **-** | **-** | **-** |
| 22 | Du et al. | 14 | 5 (35.7) | 1 (7.1) | 1 (7.1) | 7 (50.0) | 4 (28.6) | **-** | **-** | **-** | 1 (7.1) | 1 (7.1) | **-** | **-** | **-** | **-** | **-** | **-** | **-** |
| 28 | Tang et al. | 26 | **-** | 3 (11.5) | 3 (8.3) | 12 (46.2) | **-** | **-** | **-** | 7 (26.9) | **-** | **-** | **-** | **-** | **-** | **-** | **-** | **-** | **-** |
| 29 | Peng et al. | 35 | **-** | **-** | **-** | **-** | **-** | **-** | **-** | **-** | **-** | **-** | **-** | **-** | 1 (1.3) | **-** | 3 (3.9) | **-** | **-** |
| 30 | Wu et al. | 74 | 2 (2.7) | **-** |  |  | **-** | **-** | **-** | 5 (35.7) | **-** | **-** | **-** | **-** | 3 (15.7) | 16 (84.2) | 3 (15.8) | 3 (15.8) | **-** |
| 31 | Liu et al. | 248 | **-** | **-** |  |  | **-** | **-** | **-** | **-** | **-** | 13 (5.2) | 33 (13.3) | **-** | 1 (0.4) | 24 (9.7) | **-** | **-** | **-** |
| 32 | Yu et al. | 82 | 36 (43.9) | 11 (13.4) | 23 (28.0) | 15 (18.3) | **-** | **-** | **-** | **-** | 1 (2.0) | 6 (17.1) | 13 (37.1) | **-** | 1 (4.4) | 17 (42.5) | 3 (8.8) | 2 (2.4) | 1 (2.9) |
| 33 | Zhang et al. | 34 | **-** | **-** | **-** | 28 (82.4) | **-** | **-** | **-** | **-** | **-** | **-** | **-** | 3 (8.8) | 6 (17.7) | 9 (26.5) | 2 (5.9) | 2 (5.8) | 1 (2.9) |
| 34 | Tan et al. | 10 | **-** | **-** | 2 (20.0) | 1 (10.0) | **-** | **-** | **-** | **-** | **-** | **-** | **-** | **-** | **-** | 3 (30.0) | **-** | **-** | **-** |
| 35 | Xu et al. | 32 | **-** | 6 (18.8) | 1 (3.1) | 11 (37.0) | **-** | **-** | 4 (12.5) | **-** | **-** | **-** | **-** | **-** | **-** | **-** | **-** | **-** | **-** |
| 37 | Liu et al. | 91 | 54 (59.3) | **-** | **-** | 33 (36.3) |  | **-** | **-** | **-** | **-** | **-** | **-** | **-** | **-** | 3 (3.3) | 1 (1.1) | **-** | 2 (2.2) |
| 38 | Ji et al. | 4 | **-** | 1 (25.0) | 1 (25.0) | 2 (50.0) | 2 (50.0) | **-** | **-** | **-** | **-** | **-** | **-** | **-** | 2 (50.0) | 1 (25.0) | 1 (25.0) | **-** | **-** |
| 39 | Wang et al. | 31 | 1 (3.6) | 6 (22.2) | 6 (22.2) | 2 (7.7) | 4 (14.8) | **-** | **-** | 4 (19.0) | **-** | **-** | **-** | **-** | **-** | **-** | **-** | **-** | **-** |
| 40 | Zhou et al. | 9 | **-** | **-** | 4 (44.4) | 3 (60.0) |  | **--** | **-** | **-** | **-** | **-** |  | **-** | **-** | **-** | 1 (16.7) | **-** | **-** |
| 41 | Ma et al. | 115 | **-** | 11 (9.6) | **-** | **-** | **-** | 2 (1.7) | 3 (2.6) | **-** | **-** | **-** | **-** | **-** | **-** | 4 (8.9) | **-** | **-** | 1 (2.2) |
| 42 | Tan et al. | 13 | **-** | **-** | **-** | **-** | **-** | **-** | **-** | 3 (23.0) | **-** | **-** | **-** | **-** | **-** | **-** | **-** | **-** | **-** |
| 43 | Feng et al. | 15 | **-** | **-** | **-** | **-** | **-** | **-** | **-** | **-** | **-** | **-** | **-** | **-** | **-** | **-** | **-** | **-** | **-** |
| 46 | Zhang et al. | 10 | **-** | **-** | **-** | 7 (70.0) | **-** | **-** | **-** | **-** | **-** | **-** | **-** | **-** | **-** | **-** | **-** | **-** | **-** |
| 47 | Wu et al. | 23 |  | **-** | **-** | **-** | **-** | **-** | **-** | 1 (16.7) | **-** | **-** | **-** | 1 (6.3) | **-** | 2 (15.4) | **-** | **-** | **-** |
| 48 | Li et al. | 30 | **-** | **-** | **-** | **-** | **-** | **-** | **-** | **-** | **-** | **-** | **-** | **-** | **-** | 10 (33.3) | **-** | 1 (3.3) | 1 (3.3) |
| 49 | Xiong et al. | 6 | **-** | **-** | **-** | **-** | **-** | **-** | **-** | **-** | **-** | **-** | **-** | **-** | **-** | **-** | **-** | **-** | **-** |
| 50 | Zheng et al. | 9 | **-** | **-** | **-** | 5 (55.6) | **-** | **-** | **-** | **-** | **-** | **-** | **-** | **-** | **-** | **-** | **-** | **-** | **-** |
| 51 | Ma et al. | 22 | **-** | **-** | **-** | **-** | **-** | **-** | **-** | **-** | **-** | **-** | **-** | **-** | **-** | **-** | **-** | **-** | **-** |
| 52 | Chen et al. | 20 | **-** | **-** | **-** | 7 (35.0) | **-** | **-** | **-** | 3 (15.0) | **-** | **-** | **-** | **-** | **-** | **-** | **-** | **-** | **-** |
