# Supplementary material for: Epidemiological and Clinical Characteristics of COVID-19 in Children: A Systematic Review and Meta-Analysis
Source: Front Pediatr. 2020 Nov 2;8:591132. doi: 10.3389/fped.2020.591132 (PMC7667131; doi:10.3389/fped.2020.591132)
Supplement: Supplementary file 6 [file Table_6.DOCX]

**Supplementary Table 6 Imaging characteristics of the included studies on COVID-19, 2020**

| **ID** | **Author** | **N** | **n (%)** | | | | | | | |
| --- | --- | --- | --- | --- | --- | --- | --- | --- | --- | --- |
|  |  |  | **Unilateral pneumonia** | **Bilateral pneumonia** | **Ground-glass opacity** | **Pulmonary consolidation** | **Ground glass opacities and consolidation** | **Pleural effusion** | **Subpleural lesions** | **White lung** |
| 1 | Cai et al. | 10 | 4 (40) | - | 4 (40) | - | - | - | - | - |
| 2 | Hu et al. | 6 | - | - | 2 (33.3) | - | - | - | - | - |
| 3 | Zhu et al. | 10 | 3 (30) | 2 (20) | 3 (30) | - | - | - | - | - |
| 6 | Liu et al. | 6 | - | 4 (66.7) | 4 (66.7) | - | - | - | - | - |
| 8 | Liu et al. | 5 | 3 (60) | 1 (20) | 4 (80) | 1 (20) | - | - | 1 (20) | - |
| 10 | Su et al. | 9 | 1 (11.1) | - | 1 (11.1) | 1 (11.1) | - | - | 1 (11.1) | - |
| 11 | Xu et al. | 10 | - | - | 5 (50) | - | - | - | - | - |
| 12 | Li et al. | 5 | - | - | 3 (60) | - | - | - | - | - |
| 13 | Xia et al. | 20 | 6 (30) | 10 (50) | 12 (60) | 1 (5) | - | - | 20 (100) | 1 (5) |
| 14 | Liu et al. | 4 | - | - | 1 (25) | 2 (50) | - | 1 (25) | 3 (75) | - |
| 15 | Qiu et al. | 36 | - | - | 19 (52.8) | - | - | - | - | - |
| 16 | Zheng et al. | 25 | 5 (20) | 12 (48) | 1 (4) | 2 (8) | - | - | - | - |
| 17 | Sun et al. | 8 | 2 (25) | 6 (75) | 6 (75) | - | - | 1 (12.5) | - | 1 (12.5) |
| 18 | Shen et al. | 9 | 2 (22.2) | - | 2 (22.2) | - | - | - | - | - |
| 19 | Li et al. | 22 | 5 (22.7) | 15 (68.1) | 3 (13.6) | 7 (31.8) | 8 (36.4) | - | 10 (45.5) | - |
| 20 | Li et al. | 40 | 13 (32.5) | 26 (65) | - | - | - | - | - | - |
| 21 | Han et al. | 7 | - | 5 (71.4) | - | - | - | - | - | - |
| 22 | Du et al. | 14 | 5 (35.7) | 6 (42.9) | - | - | - | - | - | - |
| 24 | See et al. | 4 | - | - | 2 (50) | - | - | - | - | - |
| 25 | Lu et al. | 171 | 56 (32.7) | - | 56 (32.7) | - | - | - | 2 (1.2) | - |
| 28 | Tang et al. | 26 | 11 (42.3) | 7 (26.9) | - | - | - | - | - | - |
| 29 | Peng et al. | 35 | - | 25 (71.4) | - | - | - | - | - | - |
| 30 | Wu et al. | 74 | 21 (36.5) | 16 (21.6) | 9 (12.2) | - | - | - | 9 (12.2) | - |
| 32 | Yu et al. | 82 | 38 (46.3) | 30 (36.6) | 18 (21.9) | 3 (3.7) | - | 1 (1.2) | - | - |
| 33 | Zhang et al. | 34 | 14 (41.2) | 14 (41.2) | 28 (82.4) | - | - | - | - | - |
| 34 | Tan et al. | 10 | 4 (40) | 1 (10) | 5 (50) | - | - | - | - | - |
| 35 | Xu et al. | 32 | 8 (25) | 3 (9.4) | 8 (25) | - | - | - | - | - |
| 37 | Liu et al. | 91 | 12 (13.2) | 11 (12.1) | 11 (12.1) | - | - | - | - | - |
| 38 | Ji et al. | 4 | 1 (25) | 2 (50) | 1 (25) | - | - | - | - | - |
| 39 | Wang et al. | 31 | - | - | 9 (29.0) | - | - | - | - | - |
| 40 | Zhou et al. | 9 | 4 (44.4) | 4 (44.4) | 1 (11.1) | 1 (11.1) | 6 (66.7) | 1 (11.1) | - | - |
| 41 | Ma et al. | 115 | - | - | 49 (42.6) | - | - | 2 (1.7) | - | 2 (1.7) |
| 42 | Tan et al. | 13 | - | - | 2 (15.4) | - | - | - | - | - |
| 43 | Feng et al. | 15 | - | - | 7 (46.7) | - | - | - | - | - |
| 44 | Yang et al. | 10 | 1 (10) | 5 (50) | 1 (10) | - | - | - | - | - |
| 45 | Jiang et al. | 6 | 2 (33.3) | - | 1 (16.7) | - | - | - | - | - |
| 47 | Wu et al. | 23 | 4 (17.4) | 8 (34.8) | 9 (39.1) | 3 (13.0) | - | - | - | - |
| 48 | Li et al. | 30 | - | 7 (23.3) | 5 (16.7) | 2 (6.7) | 23 (76.7) | - | - | - |
| 49 | Xiong et al. | 6 | 2 (33.3) | - | 2 (33.3) | - | - | - | - | - |
| 50 | Zheng et al. | 9 | 6 (66.7) | - | 4 (44.4) | 1 (11.1) | 1 (11.1) | - | - | - |
| 51 | Ma et al. | 22 | 7 (31.8) | 12 (54.5) | 6 (27.3) | 4 (18.2) | 6 (27.3) | - | - | 1 (4.5) |
| 52 | Chen et al. | 20 | - | - | 13 (65) | 1 (5) | - | 1 (5) | - | - |
| 54 | Feng et al. | 5 | 1 (20) | 2 (40) | 1 (20) | - | - | - | - | - |
