# Supplementary material for: Epidemiological and Clinical Characteristics of COVID-19 in Children: A Systematic Review and Meta-Analysis
Source: Front Pediatr. 2020 Nov 2;8:591132. doi: 10.3389/fped.2020.591132 (PMC7667131; doi:10.3389/fped.2020.591132)
Supplement: Supplementary file 7 [file Table_7.DOCX]

**Supplementary Table 7 Treatment and outcomes of the included studies on COVID-19, 2020**

| **ID** | **Author** | **N** | **n (%)** | | | | | | | | | | | |
| --- | --- | --- | --- | --- | --- | --- | --- | --- | --- | --- | --- | --- | --- | --- |
|  |  |  | **Treatment** | | | | | | | | | | **Outcomes** | |
|  |  |  | **Oxygen therapy** | **Mechanical ventilation** | **Interferon-alpha** | **Ribavirin** | **Oseltamivir** | **Lopinavir/ritonavir** | **Glucocorticoids** | **Antibiotics** | **Immunoglobulin** | **Traditional Chinese medicine** | **Discharged** | **Death** |
| 1 | Cai et al. | 10 | - | - | - | - | - | - | - | 5 (50) | - | - | 10 (100) | - |
| 2 | Hu et al. | 6 | - | - | - | - | - | - | - | - | - | - | 3 (50) | - |
| 3 | Zhu et al. | 10 | 1 (10) | - | 4 (40) | - | 1 (10) | 4 (40) | - | 1 (10) | - | - | 5 (50) | - |
| 4 | CDC COVID-19 Team | 745 | - | - | - | - | - | - | - | - | - | - | - | 3(0.4) |
| 6 | Liu et al. | 6 | 1 (16.7) | - | - | 2 (33.3) | 6 (100) | - | 4 (66.7) | - | 1 (16.7) | - | 6 (100) | - |
| 8 | Liu et al. | 5 | - | - | 1 (20) | 3 (60) | - | - | - | - | - | - | - | - |
| 9 | Tagarro et al. | 41 | - | 1 (2.4) | - | - | - | - | - | - | - | - | - | - |
| 10 | Su et al. | 9 | - | - | 9 (100) | 1 (11.1) | - | - | - | - | - | - | 9 (100) | - |
| 11 | Xu et al. | 10 | - | - | 10 (100) |  | - | - | - | 1 (10) | - | - | 4 (40) | - |
| 12 | Li et al. | 5 | - | - | 2 (40) | 2 (40) | - | - | - | - | 5 (100) | 1 (20) | 3 (60) | - |
| 13 | Xia et al. | 20 | - | - | - | - | - | - | - | - | - | - | 18 (90) | - |
| 15 | Qiu et al. | 36 | 6 (16.7) | - | 36 (100) | 14 (38.9) | - | 14 (38.9) | - | - | - | - | 36 (100) | - |
| 16 | Zheng et al. | 25 | 2 (8) | 2 (8) | 12 (48) | - | - | - | 2 (8) | 13 (42) | 2 (8) | - | 1 (4) | - |
| 17 | Sun et al. | 8 | 6 (75) | 2 (25) | 8 (100) | 8 (100) | - | - | 5 (62.5) | 5 (62.5) | 4 (50) | 4 (50) | 5 (62.5) | - |
| 18 | Shen et al. | 9 | 9 (100) | - | - | - | - | 9 (100) | 1 (11.1) | 4 (44.4) | 1 (11.1) | - | 6 (66.7) | - |
| 20 | Li et al. | 40 | - | 1 (2.5) | 40 (100) | - | 20 (50) | - | 3 (7.5) | 13 (32.5) | 4 (10) | - | - | - |
| 21 | Han et al. | 7 | 2 (28.6) | - | - | - | - | - | 1 (14.3) | - | - | - | 7 (100) | - |
| 24 | See et al. | 4 | - | - | - | - | - | - | - | 1 (25) | - | - | 4 (100) | - |
| 25 | Lu et al. | 171 | - | - | - | - | - | - | - | - | - | - | 149 (87.1) | 1(0.58) |
| 28 | Tang et al. | 26 | - | - | 4 (15.4) | - | - | 1 (3.8) | - | - | - | - | 17 (65.4) | - |
| 29 | Peng et al. | 35 | - | - | - | - | - | - | - | - | - | - | 26 (74.3) | - |
| 30 | Wu et al. | 74 | - | - | - | - | - | - | 1 (1.4) | 27 (36.5) | 1 (1.4) | - | 74 (100) | - |
| 32 | Yu et al. | 82 | 5 (6.1) | 2 (2.4) | - | 82 (100) | - | - | 3 (36.6) | 70 (85.4) | 3 (36.6) | 10 (12.2) | 60 (73.2) | - |
| 33 | Zhang et al. | 34 | 3 (8.8) | - | 28 (82.4) | 28 (82.4) | - | - | 5 (14.7) | 30 (88.2) | - | - | - | - |
| 34 | Tan et al. | 10 | - | - | - | - | - | - | - | 1 (10) | - | - | 10 (100) | - |
| 35 | Xu et al. | 32 | - | - | 30 (93.8) | 3 (9.4) | - | 15 (46.9) | - | 3 (9.4) | - | - | - | - |
| 36 | Shekerdemian et al. | 48 | 6 (12.5) | 18 (37.5) | - | - | - | - | - | 8 (16.7) | - | - | 31 (64.6) | 2(4.2) |
| 37 | Liu et al. | 91 | - | - | - | - | - | - | - | - | - | - | 73 (80.2) | - |
| 38 | Ji et al. | 4 | - | - | 4 (100) | 4 (100) | - | - | 3 (75) | 2 (50) | - | 3 (75) | 4 (100) | - |
| 39 | Wang et al. | 31 | - | - | 10 (32.3) | - | 1 (3.2) | - | 2 (6.5) | 6 (19.4) | - | 9 (29.0) | 24 (77.4) | - |
| 40 | Zhou et al. | 9 | - | - | 9 (100) | - | - | 6 (66.7) | - | - | - | - | - | - |
| 42 | Tan et al. | 13 | - | - | 10 (76.9) | 6 (46.2) | - | 12 (92.3) | - | - | - | - | 13 (100) | - |
| 43 | Feng et al. | 15 | - | - | - | - | - | - | - | - | - | - | 5 (33.3) | - |
| 44 | Yang et al. | 10 | - | - | - | - | - | - | - | - | - | - | 6 (60) | - |
| 46 | Zhang et al. | 10 | - | - | - | - | - | - | - | - | - | - | 2 (20) | - |
| 47 | Wu et al. | 23 | 4 (17.4) | - | 17 (73.9) | 5 (21.7) | 5 (21.7) | 6 (26.1) | - | 7 (30.4) | - | 9 (39.1) | 23 (100) | - |
| 51 | Ma et al. | 22 | - | - | - | - | - | - | - | - | - | - | 5 (22.7) | - |
| 52 | Chen et al. | 20 | - | - | 20 (100) | - | - | 15 (75) | - | - | - | - | 16 (80) | - |
| 53 | Yang et al. | 11 | - | - | - | - | - | - | - | - | - | - | 11 (100) | - |
| 54 | Feng et al. | 5 | - | - | - | - | - | - | - | - | - | - | 3 (60) | - |
