# Supplementary material for: Epidemiological and Clinical Characteristics of COVID-19 in Children: A Systematic Review and Meta-Analysis
Source: Front Pediatr. 2020 Nov 2;8:591132. doi: 10.3389/fped.2020.591132 (PMC7667131; doi:10.3389/fped.2020.591132)
Supplement: Supplementary file 8 [file Table_8.DOCX]

**Supplementary Table 8 Case reports included in the study.**

| **Author** | **Journal** | **Date (MM/DD)** | **Country** | **Number** | | | **Age/mean (months)** |
| --- | --- | --- | --- | --- | --- | --- | --- |
|  |  |  |  | **ALL** | **M** | **F** |  |
| Canarutto et al (1) | Pediatr Pulmonol | 03/19 | Italy | 1 | 1 | 0 | 1.1 |
| Kam et al (2) | Clin Infect Dis | 02/28 | China | 1 | 1 | 0 | 6 |
| Kamal.i et al (3) | Infect Dis (Lond) | 04/01 | Iran | 1 | 1 | 0 | 0.5 |
| Li et al (4) | Pediatr Pulmonol | 03/05 | China | 2 | 1 | 1 | 48 |
| Lin et al (5) | Pediatr Pulmonol | 03/24 | China | 1 | 0 | 1 | 84 |
| Park et al (6) | J Korean Med Sci | 03/23 | Korea | 1 | 0 | 1 | 120 |
| Wang et al (7) | Clin Infect Dis | 03/12 | China | 1 | 1 | 0 | 0 |
| Cui et al (8) | J Infect Dis | 03/17 | China | 1 | 0 | 1 | 1.8 |
| Wu et al (9) | Graefes Arch Clin Exp Ophthalmol | 04/24 | China | 1 | 1 | 0 | 34 |
| Pan et al (10) | Lancet Infect Dis | 02/19 | China | 1 | 1 | 0 | 36 |
| Wang et al (11) | Pediatr Infect Dis | 03/23 | China | 1 | 1 | 0 | 96 |
| Hai et al (12) | Lancet Child Adolesc Health | 03/23 | Vietnam | 1 | 0 | 1 | 3 |
| Mansour et al (13) | Cureus | 04/03 | Lebanon | 1 | 0 | 1 | 16 |
| Michal. et al (14) | Clin Infect Dis | 04/17 | USA | 2 | 1 | 0 | 1.35 |
| Al.varo et al (15) | NEJM | 04/22 | USA | 1 | 1 | 0 | 0.7 |
| Wang et al (16) | Chin Med Care Repository | 04/09 | China | 1 | 1 | 0 | 36 |
| Zhang et al (17) | Chin J Pediatr | 02/07 | China | 1 | 0 | 1 | 3 |
| Wang et al (18) | Chin J Evid Based Pediatr | 02/17 | China | 1 | 0 | 1 | 19.2 |
| Zeng et al (19) | Chin J Pediatr | 04/01 | China | 1 | 1 | 0 | 0.6 |
| Liu et al (20) | Med J Wuhan Univ | 05/01 | China | 1 | 1 | 0 | 108 |
| Chen et al (21) | Chin J Pediatr | 02/08 | China | 1 | 1 | 0 | 12 |
| Cai et al (22) | Chin J Pediatr | 01/31 | China | 1 | 1 | 0 | 84 |
| Wang et al (23) | Chin J Contemp Pediatr | 03/01 | China | 1 | 1 | 0 | 0.6 |
| Deng et al (24) | Chin Pediatr Emerg Med | 02/01 | China | 2 | 1 | 1 | 96 |
| Xiong et al (25) | Chin Pediatr Emerg Med | 02/14 | China | 1 | 1 | 0 | 60 |
| Chen et al (26) | Chin J Hematol | 02/19 | China | 1 | 1 | 0 | 96 |
| Shui et al (27) | Chin Med Care Repository | 03/06 | China | 1 | 0 | 1 | 7 |
| Wei et al (28) | Acad J Chin PLA Med Sch | 04/03 | China | 1 | 1 | 0 | 1.1 |
| Zhang et al (29) | Chin J Contemp Pediatr | 03/01 | China | 2 | 0 | 2 | 12 |
| Zhao et al (30) | Zhejiang Med J | 02/16 | China | 1 | 1 | 0 | 156 |
| Yu et al (31) | Chin J Evid Based Pediatr | 03/10 | China | 1 | 1 | 0 | 0.7 |
| Li et al (32) | J Chongqing Med Univ | 03/31 | China | 1 | 0 | 1 | 36 |
| Sha et al (33) | Henan J Prev Med | 02/28 | China | 1 | 1 | 0 | 60 |
| Jiao et al (34) | J Pediatr Pharm | 03/14 | China | 1 | 0 | 1 | 7 |
| Quan et al (35) | Chin Pediatr Emerg Med | 02/28 | China | 1 | 0 | 1 | 48 |
| Cheng et al (36) | Chin J TCM WM Crit Care | 02/18 | China | 2 | 1 | 1 | 11.5 |
| Yang et al (37) | J Wenzhou Med Univ | 04/22 | China | 1 | 0 | 1 | 120 |
| Ji et al (38) | World J Pediatr | 03/16 | China | 2 | 2 | 0 | 144 |
| Zhang et al (39) | J Med Virol | 03/23 | China | 3 | 3 | 0 | 92 |
| Zheng et al (40) | Curr Med Sci | 03/25 | China | 2 | 2 | 0 | 12 |
| Lou et al (41) | J Paediatr Child Health | 03/22 | China | 3 | 1 | 2 | 58 |
| Mogharab et al (42) | J Formos Med Assoc | 04/13 | Iran | 1 | - | - | 2.5 |

**References**

1. Canarutto D, Priolo A, Russo G, Pitea M, Vigone MC, Barera G. COVID-19 infection in a paucisymptomatic infant: Raising the index of suspicion in epidemic settings. Pediatr pulmonol (2020) 55(6): E4-e5. doi:10.1002/ppul.24754

2. Kam KQ, Yung CF, Cui L, et al. A well infant with coronavirus disease 2019 (COVID-19) with high viral load. Clin Infect Dis (2020) 71(15):847-849. doi:10.1093/cid/ciaa201

3. Kamali Aghdam M, Jafari N, Eftekhari K. Novel coronavirus in a 15-day-old neonate with clinical signs of sepsis, a case report. Infect Dis (Lond) (2020)52(6):427-429. doi:10.1080/23744235.2020.1747634

4. Li Y, Guo F, Cao Y, Li L, Guo Y. Insight into COVID-2019 for pediatricians. Pediatr Pulmonol (2020)55(5): E1-E4. doi:10.1002/ppul.24734

5. Lin JL, Duan J, Tan TD, Fu Z, Dai JH. The isolation period should be longer: Lesson from a child infected with SARS-CoV-2 in Chongqing, China. Pediatr Pulmonol (2020)55(6): E6-E9. doi:10.1002/ppul.24763

6. Park JY, Han MS, Park KU, Kim JY, Choi EH. First pediatric case of coronavirus disease 2019 in Korea. J Korean Med Sci (2020)35(11): e124. doi:10.3346/jkms.2020.35. e124

7. Wang S, Guo L, Chen L, et al. A case report of neonatal COVID-19 infection in China. Clin Infect Dis (2020) 71(15):853-857. doi:10.1093/cid/ciaa225

8. Cui Y, Tian M, Huang D, et al. A 55-Day-Old female infant infected with COVID 19: Presenting with pneumonia, liver injury, and heart damage. J Infect Dis (2020)221(11):1775-1781. doi:10.1093/infdis/jiaa113

9. Wu P, Liang L, Chen CB, Nie SQ. A child confirmed COVID-19 with only symptoms of conjunctivitis and eyelid dermatitis. Graefes Arch Clin Exp Ophthalmol (2020)258(7):1565-1566. doi:10.1007/s00417-020-04708-6

10. Pan X, Chen D, Xia Y, et al. Asymptomatic cases in a family cluster with SARS-CoV-2 infection. Lancet Infect Dis (2020)20(4):410-411. doi:10.1016/s1473-3099(20)30114-6

11. Wang H, Li Y, Wang F, Du H, Lu X. Rehospitalization of a recovered coronavirus disease 19 (COVID-19) child with positive nucleic acid detection. Pediatr Infect Dis (2020)39(6): e69-e70. doi:10.1097/inf.0000000000002690

12. Le HT, Nguyen LV, Tran DM, et al. The first infant case of COVID-19 acquired from a secondary transmission in Vietnam. Lancet Child Adolesc Health (2020)4(5):405-406. doi:10.1016/s2352-4642(20)30091-2

13. Mansour A, Atoui R, Kanso K, Mohsen R, Fares Y, Fares J. First case of an infant with COVID-19 in the Middle East. Cureus (2020)12(4). doi:10.7759/cureus.7520

14. Paret M, Lighter J, Madan RP, Raabe VN, Shust GF, Ratner AJ. SARS-CoV-2 infection (COVID-19) in febrile infants without respiratory distress. Clin Infect Dis [Preprint] (2020). Available at: https:// doi.org.10.1093/cid/ciaa452 (Accessed April 17, 2020).

15. Munoz AC, Nawaratne U, McMann D, Ellsworth M, Meliones J, Boukas K. Late-onset neonatal sepsis in a Patient with Covid-19. N Engl J Med (2020)382(19): e49. doi:10.1056/NEJMc2010614

16. Wang L, Li YT, Wang F. First case of COVID-19 in children in Nanyang. Chin Med Care Repository (2020). doi:10.3760/cma.j.cmcr.2020.e00018

17. Zhang YH, Lin DJ, Xiao MF, et al. 2019-novel coronavirus infection in a three-month-old baby. Chin J Pediatr (2020)58(0):E006. doi:10.3760/cma.j.issn.0578-1310

18. Wang JY, Wang YJ, Zhou HQ, et al. A case report of children with 2019 novel coronavirus infection. Chin J Evid Based Pediatr (2020). doi:10.3969/j.issn.1673-5501

19. Zeng LK, Tao XW, Yuan WH, Wang J, Liu X, Liu ZS. First case of neonate with COVID-19 in China. Chin J Pediatr (2020)58(4):279-280. doi:10.3760/cma.j.cn112140‑20200212‑00081

20. Liu M, Wan X, Tu XY, et al. Family cluster of child SARA-CoV-2 infections: a case report. Med J Wuhan Univ (2020)41(03):362-365. doi:10.14188/j.1671‑8852.2020.0078

21. Chen F, Liu ZS, Zhang FR, et al. First case of severe childhood novel coronavirus pneumonia in China. Chin J Pediatr (2020)58(3):179-182. doi:10.3760/cma.j.issn.0578-1310.2020.03.000

22. Cai JH, Wang XS, Ge YL, et al. First case of 2019 novel coronavirus infection in children in Shanghai. Chin J Pediatr (2020)58(2):86-87. doi:10.3760/cma.j.issn.0578-1310.2020.02.002

23. Wang J, Wang D, Chen GC, Tao XW, Zeng LK. SARS-CoV-2 infection with gastrointestinal symptoms as the first manifestation in a neonate. Chin J Contemp Pediatr (2020)22(3):211-214 doi:10.7499/j.issn.1008-8830.2020.03.006

24. Deng HL, Zhang YF, Wang Y, Li FY. Two cases of new coronavirus infection in children. Chin Pediatr Emerg Med (2020)27(2):81-83.

25. Xiong DX, Jiang JY, Feng Y, et al. Two cases of new coronavirus pneumonia in children. Chin Pediatr Emerg Med (2020).

26. Chen Z, Xiong H, Li JX, et al. COVID-19 with post-chemotherapy agranulocytosis in childhood acute leukemia: a case report. Chin J Hematol (2020)41(0):E004.

27. Shui LL, Chen YJ, Xiao KH, Wang JB. Clinical feature of the first case of COVID-19 in infant with familial cluster outbreak in Chongqing. Chin Med Care Repository (2020) doi:10.3760/cma.j.cmcr.2020.e00011

28. Wei W, Yuan CL, Liu X, Zheng M. One case of asymptomatic infection of infants with new coronavirus with fecal nucleic acid test. Acad J Chin PLA Med Sch (2020):1-3.

29. Zhang GX, Zhang AM, Huang L, et al. Twin girls infected with SARS-CoV-2. Chin J Contemp Pediatr(2020)22(3):221-225. doi:10.7499/j.issn.1008-8830.2020.03.008

30. Zhao RH, Shen XM, Xu KJ, Sheng JF. A case of children infected with new coronavirus pneumonia. Zhejiang Med J (2020). doi:10.12056/j.issn.1006-2785.2020.42.3.2020-337

31. Yu ZY, Xue W, Feng YJ, et al. Transport, diagnosis and treatment of a newborn with severe SARS-CoV-2 infection: A case report. Chin J Evid Based Pediatr (2020). doi:10.3969/j.issn.1673-5501.2020.01.009

32. Li X, Zhang T, Tang M, Wang YP. COVID-19 pneumonia report of the first child in Yunnan. J Chongqing Med Univ (2020):1-2. doi:10.13406/j.cnki.cyxb.002419

33. Sha GJ. A case report of children infected with novel coronavirus and influenza virus. Henan J Prev Med (2020). doi:10.13515/j.cnki.hnjpm.1006-8414.2020.05.002

34. Jiao SF, Yang W, Pan DC. Analysis of medication for a case of mild new coronavirus pneumonia infant. J Pediatr Pharm (2020)26(04):65-66. doi:10.13407/j.cnki.jpp.1672-108X.2020.04.021

35. Quan SD, Li X, Ding DS, Sun SY, Sun GL. A case of novel coronavirus infection in children in Liaoning. Pediatr Emerg Med (2020) (00):E005-E005.

36. Cheng XF, Zhang FR, Xiong RH, et al. A case report of two children with corona virus disease 2019 complicated with diffuse intravascular coagulation. Chin J TCM WM Crit Care (2020). doi：10.3969/j.issn.1008-9691.2020.01.013

37. Yang LL, Ye PP, Ge HZ, Yan ZH, He JW. One case of mild COVID-19 with multiple hematuria, repeated anal swabs or fecal nucleic acid test. J Wenzhou Med Univ(2020):1-5.

38. Ji LN, Chao S, Wang YJ, et al. Clinical features of pediatric patients with COVID-19: a report of two family cluster cases. World J Pediatr (2020):1-4. doi:10.1007/s12519-020-00356-2.

39. Zhang T, Cui X, Zhao X, et al. Detectable SARS-CoV-2 viral RNA in feces of three children during recovery period of COVID-19 pneumonia. J Med Virol (2020)92(7):909-914. doi:10.1002/jmv.25795

40. Zheng F, Liao C, Fan QH, et al. Clinical Characteristics of Children with Coronavirus Disease 2019 in Hubei, China. Curr Med Sci (2020)40(2):275-280. doi:10.1007/s11596-020-2172-6

41. Lou XX, Shi CX, Zhou CC, Tian YS. Three children who recovered from novel coronavirus 2019 pneumonia. J Paediatr Child Health (2020)56(4):650-651. doi:10.1111/jpc.14871

42. Mogharab V, Pasha AMK, Javdani F, Hatami N. The first case of COVID-19 infection in a 75-day-old infant in Jahrom City, south of Iran. J Formos Med Assoc (2020)119(5):995-997. doi:10.1016/j.jfma.2020.03.015
