# Supplementary material for: Epidemiological and Clinical Characteristics of COVID-19 in Children: A Systematic Review and Meta-Analysis
Source: Front Pediatr. 2020 Nov 2;8:591132. doi: 10.3389/fped.2020.591132 (PMC7667131; doi:10.3389/fped.2020.591132)
Supplement: Supplementary file 9 [file Data_Sheet_1.PDF]

Supplementary Figure 1

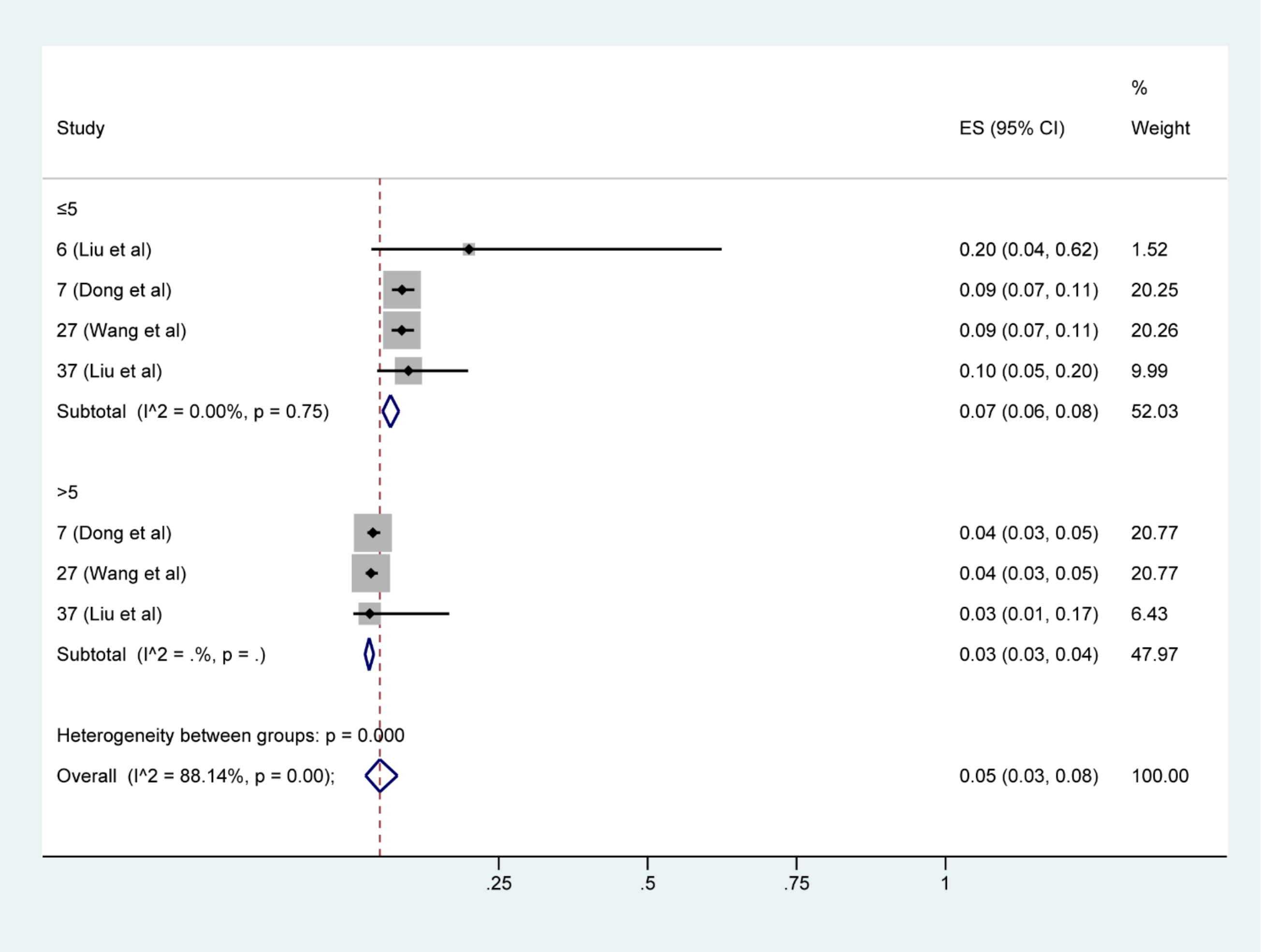

Supplementary Figure 1. Forest plot of the meta-analysis of disease severity (severe, or critical infection) in relation to age stratified by less than 5 years old and more than 5 years old. Individual studies are represented by the rate and 95% confidence interval (CI). The diamonds and horizontal lines indicate the corresponding rates and 95% CIs. The size of the gray area reflects the study-specific statistical weight. The vertical red dashed line represents the estimated combined effect.
